# The *Oryza sativa* Regulator HDR1 Associates with the Kinase OsK4 to Control Photoperiodic Flowering

**DOI:** 10.1371/journal.pgen.1005927

**Published:** 2016-03-08

**Authors:** Xuehui Sun, Zhiguo Zhang, Jinxia Wu, Xuean Cui, Dan Feng, Kai Wang, Ming Xu, Li Zhou, Xiao Han, Xiaofeng Gu, Tiegang Lu

**Affiliations:** Biotechnology Research Institute/National Key Facility for Genetic Resources and Gene Improvement, The Chinese Academy of Agricultural Sciences, Beijing, P. R. China; The University of North Carolina at Chapel Hill, UNITED STATES

## Abstract

Rice is a facultative short-day plant (SDP), and the regulatory pathways for flowering time are conserved, but functionally modified, in *Arabidopsis* and rice. *Heading date 1* (*Hd1*), an ortholog of *Arabidopsis CONSTANS* (*CO*), is a key regulator that suppresses flowering under long-day conditions (LDs), but promotes flowering under short-day conditions (SDs) by influencing the expression of the florigen gene *Heading date 3a* (*Hd3a*). Another key regulator, *Early heading date 1* (*Ehd1*), is an evolutionarily unique gene with no orthologs in *Arabidopsis*, which acts as a flowering activator under both SD and LD by promoting the rice florigen genes *Hd3a* and *RICE FLOWERING LOCUST 1* (*RFT1*). Here, we report the isolation and characterization of the flowering regulator Heading Date Repressor1 (HDR1) in rice. The *hdr1* mutant exhibits an early flowering phenotype under natural LD in a paddy field in Beijing, China (39°54'N, 116°23'E), as well as under LD but not SD in a growth chamber, indicating that *HDR1* may functionally regulate flowering time *via* the photoperiod-dependent pathway. *HDR1* encodes a nuclear protein that is most active in leaves and floral organs and exhibits a typical diurnal expression pattern. We determined that HDR1 is a novel suppressor of flowering that upregulates *Hd1* and downregulates *Ehd1*, leading to the downregulation of *Hd3a* and *RFT1* under LDs. We have further identified an HDR1-interacting kinase, OsK4, another suppressor of rice flowering under LDs. *OsK4* acts similarly to *HDR1*, suppressing flowering by upregulating *Hd1* and downregulating *Ehd1* under LDs, and OsK4 can phosphorylate HD1 with HDR1 presents. These results collectively reveal the transcriptional regulators of *Hd1* for the day-length-dependent control of flowering time in rice.

## Author Summary

In rice, flowering time affects the potential yield, the growing season and regional adaptability. Change in day length is a key seasonal cue for regulating flowering time in rice, a facultative short-day (SD) plant. The photoperiodic pathway of rice contains the evolutionarily conserved *Hd1*-*Hd3a* module, which is homologous to the *CO*-*FT* module in the long-day (LD) plant *Arabidopsis*. In this work, we cloned a novel gene, *HDR1*, that activates *Hd1* and represses *Ehd1*, thereby down-regulating the florigen genes *Hd3a* and *RFT1* to postpone rice flowering. A protein associated with HDR1, OsK4, was also identified, and the resulting complex can interact with HD1 to phosphorylate HD1. We conclude that HDR1 is a novel transcriptional regulator of *Hd1* that plays a crucial role in regulating flowering time *via* the photoperiodic pathway in rice.

## Introduction

The ability of plants to reproduce during the appropriate season enables them to adapt to environmental changes in day length and temperature and requires precise monitoring of environmental and endogenous signals [1–5]. These external and internal signals comprise a complex regulatory network that includes the aging, autonomous, vernalization, photoperiod, gibberellin, and ambient temperature pathways [6,7]. This network allows plants to grow at different latitudes and altitudes and during different seasons [3,8,9]. The basis for this complex network is the control of photoperiod-dependent flowering, which involves processes such as day-length measurement in leaves, the generation of mobile signals called florigens, the transport of florigens from leaves to the shoot apex, and the perception of florigens at the shoot apical meristem to initiate floral evocation [10,11].

The molecular mechanisms that regulate plant flowering time *via* the photoperiodic pathway have been extensively studied in *Arabidopsis*, a long-day plant (LDP), and in rice, a short-day plant (SDP) [2,6,11–13]. In *Arabidopsis*, the *GIGANTEA* (*GI*)-*CONSTANS* (*CO*)-*FLOWERING LOCUS T* (*FT*) transcriptional regulatory pathway has been characterized. *GI* integrates signals from photoreceptors and the circadian clock [14] and, as a regulator of transcription, activates *CO*, which in turn promotes *FT* expression. FT functions as a florigen and coordinates with SUPPRESSOR OF OVER-EXPRESSION OF CONSTANT 1 (SOC1) to promote flowering [15,16].

Rice is a facultative SDP. Thus, short-day conditions (SDs) promote flowering in rice plants, and multiple flowering genes prevent flowering under long-day conditions (LDs) [3,13]. Phylogenetic analyses and functional studies have revealed that the rice orthologs of *GI*, *CO*, and *FT* are *OsGI*, *Heading date 1* (*Hd1*), and *Heading date 3a* (*Hd3a*)/ *RICE FLOWERING LOCUS T1* (*RFT1*), respectively. The pathway regulating flowering time is conserved in *Arabidopsis* and rice, but the functions of specific genes differ [17–22]. Similar to *Arabidopsis GI*, *OsGI* upregulates *Hd1*, which suppresses flowering under LDs but promotes flowering under SD by influencing the expression of *Hd3a* [18,19,23–25]. *Hd3a* is also modulated by *Early heading date 1* (*Ehd1*), which encodes a B-type response regulator that functions independent of *Hd1* [26]. *Ehd1* is an evolutionarily unique gene that does not have any ortholog in *Arabidopsis* and that functions as a flowering activator under both SDs and LDs by promoting the rice florigens*Hd3a* and *RFT1* [20,21,26]. Several flowering regulators involved in this *Ehd1* pathway have been identified [27–38]. Conversely, *OsMADS51*, which is controlled by *OsGI*, serves as a flowering activator upstream of *Ehd1* under SD [27]. *OsID1/Ehd2*/*RID1*, hereafter referred to a as *Early heading date 2* (*Ehd2*), and *Early heading date 3* (*Ehd3*) promote flowering under both SDs and LDs by inducing the expression of *Ehd1* [28–30]. *OsMADS50* and *OsMADS56* antagonistically regulate LD-dependent flowering in rice [31,32]. *OsLFL1* (*Oryza sativa LEC2 and FUSCA3 Like 1*), an *Ehd1* promoter-binding B3-type transcription factor, inhibits *Ehd1* expression to repress rice flowering only under LD [33]. *Ehd1* and *Hd3a* are suppressed by *Ghd7* (*Grain number*, *plant height and heading date 7*) only under LDs, resulting in the late flowering of rice plants under LD [34]. *DTH8* (*QTL for days to heading on chromosome 8*), a major quantitative trait locus (QTL) that regulates grain productivity, plant height, and heading date in rice, is also a strong repressor of *Ehd1* and accounts for late flowering in rice under LDs [35,36]. *Ehd3*, which encodes a plant homeodomain finger-containing protein, is a critical promoter of rice flowering both under SDs and LDs by regulating the expression of *Ehd1* [37]. *Early heading date 4* (*Ehd4*) encodes a novel CCCH-type zinc-finger protein that promotes flowering only under natural LDs (NLD) [38]. Although several regulatory factors have been characterized in *Hd1*-dependent and *Ehd1*-dependent pathways in rice, whether there is an uncharacterized regulatory factor involved in these two pathways in rice is unknown.

In this study, we reported the identification of an early flowering rice mutant, and the cloning of *HDR1 (**H**eading* *D**ate* *R**epressor 1*), a novel gene encoding a 210-amino-acid protein with a molecular weight of ~23 kD. *HDR1* encodes a ubiquitous protein in plants, and the moss ortholog *PpSKI* encodes a putative kinase ligand that is important for the recognition of and adaptation to conditions of limited energy during plant development [39,40]. Expression and functional analyses of *HDR1* revealed that it activates *Hd1* and represses *Ehd1*, thereby downregulating the florigen genes *Hd3a* and *RFT1* to postpone rice flowering. An HDR1-interacting kinase protein encoded by *OsK4* was also identified, which functions to phosphorylate HD1 to regulate flowering time in rice.

## Results

### Identification and characterization of the early flowering mutant *hdr1*

**Fig 1:**
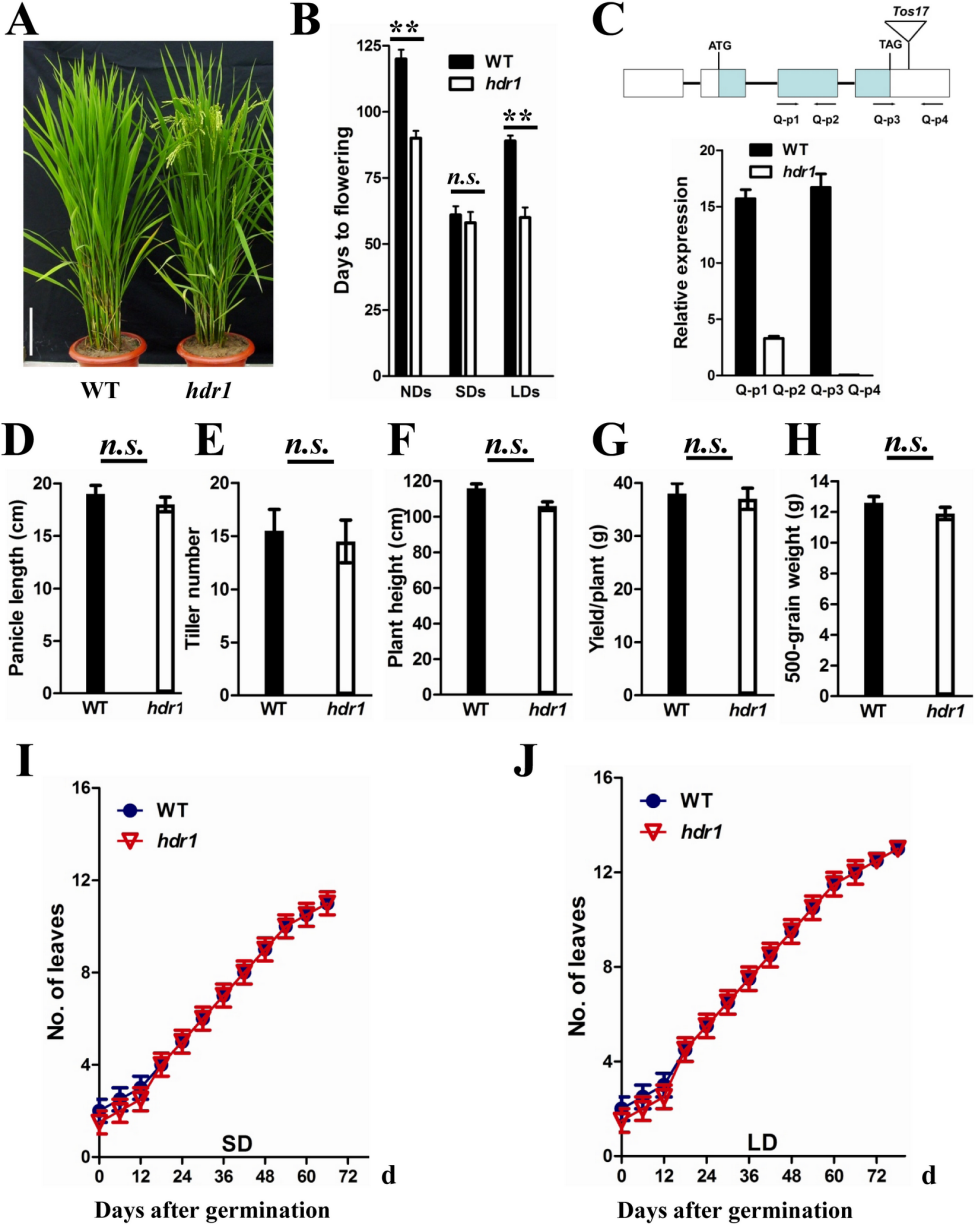
Phenotypes of the *hdr1* mutant. (A) The early flowering phenotype of *hdr1* mutant in a paddy field (right). The Nipponbare WT (left). Bar = 20 cm. (B) Flowering time of *hdr1* and WT plants under different day-length conditions (n = 12). NDs, natural day conditions; SDs, short day conditions; LDs, long day conditions. (C) mRNA expression analysis of *HDR1* using real-time PCR in WT and *hdr1* mutant. Mean values ±s.d. were obtained from three technical replicates and two biological replicates. (D) to (H) Comparisons of plant height, tilling number, panicle length, 500-grain weight, and yield per plant between WT and *hdr1* plants. The values presented are the mean ± s.d. (n = 15). Double asterisks indicate statistically significant differences in the means between WT and *hdr1* according to a two-tailed Student’s t test (p<0.01), and n.s. indicates no significant difference. (I) and (J) *hdr1* plants had the same leaf-emergence rate as WT under both SDs and LDs. The values presented are the mean ± s.d. (n = 12). s.d.: standard deviations.

We previously reported the development and characterization of T-DNA-mutagenized rice populations [41–43]. An average of 2.8 copies of T-DNA and 2.1 copies of newly transposed *Tos17* were integrated into the rice genome during transformation process. Systematic characterization of the mutant populations led to the identification of early and late flowering mutants, and the sequences flanking the T-DNA/*Tos17* insertion sites in the mutants were amplified [41–43]. One mutant, named *hdr1*, flowered approximately 30 days earlier than wild-type (WT, *Oryza sativa* var. Nipponbare) plants following growth under NLDs (Fig 1A) in a paddy field in Beijing, China (39°54'N, 116°23'E). The phenotypes of WT and *hdr1* plants were assessed under natural-day field conditions (NDs) in Beijing and under LDs (14 h of light/10 h of dark) and SDs (10 h of light/14 h of dark) conditions in a controlled growth chamber. Under NDs and LDs, the *hdr1* plants flowered approximately 30 days earlier than the WT plants, whereas no significant difference was observed under SDs (Fig 1B). These data suggested that *HDR1* might participate in the regulation of photoperiodic flowering in rice.

A genetic analysis revealed that a newly transposed copy of *Tos17* that was inserted into the 3'-untranslated region (UTR; 22 nt after the stop codon) of a rice gene (LOC_Os02 g55080 in the TIGR database [http://rice.plantbiology.msu.edu]) co-segregated with the early flowering phenotype of *hdr1*. *HDR1* expression was analyzed using quantitative real-time PCR (qPCR) in WT and *hdr1* plants using two pairs of primers based on the sequences of exon 2 (Q-p1 and Q-p2) and the 3’-UTR (Q-p3 and Q-p4). Primers Q-p1 and Q-p2 indicated that *HDR1* expression was dramatically decreased in *hdr1*, and expression was only very weakly detected using primers Q-p3 and Q-p4, indicating that a low level of *HDR1* mRNA was produced in the *hdr1* plants (Fig 1C). The mature *hdr1* plants were slightly shorter and had similar numbers of tillers (Fig 1D and 1E). No obvious difference in panicle length, 500-grain weight, or yield per plant was detected, indicating that the fertility levels of the mutant and WT plants were similar (Fig 1F–1H). Notably, the leaf-emergence rate of *hdr1* was similar to that of WT under both SDs and LDs (Fig 1I and 1J), indicating that the early flowering phenotype was not caused by an increase in growth rate.

### *HDR1* controls photoperiodic flowering in rice

**Fig 2:**
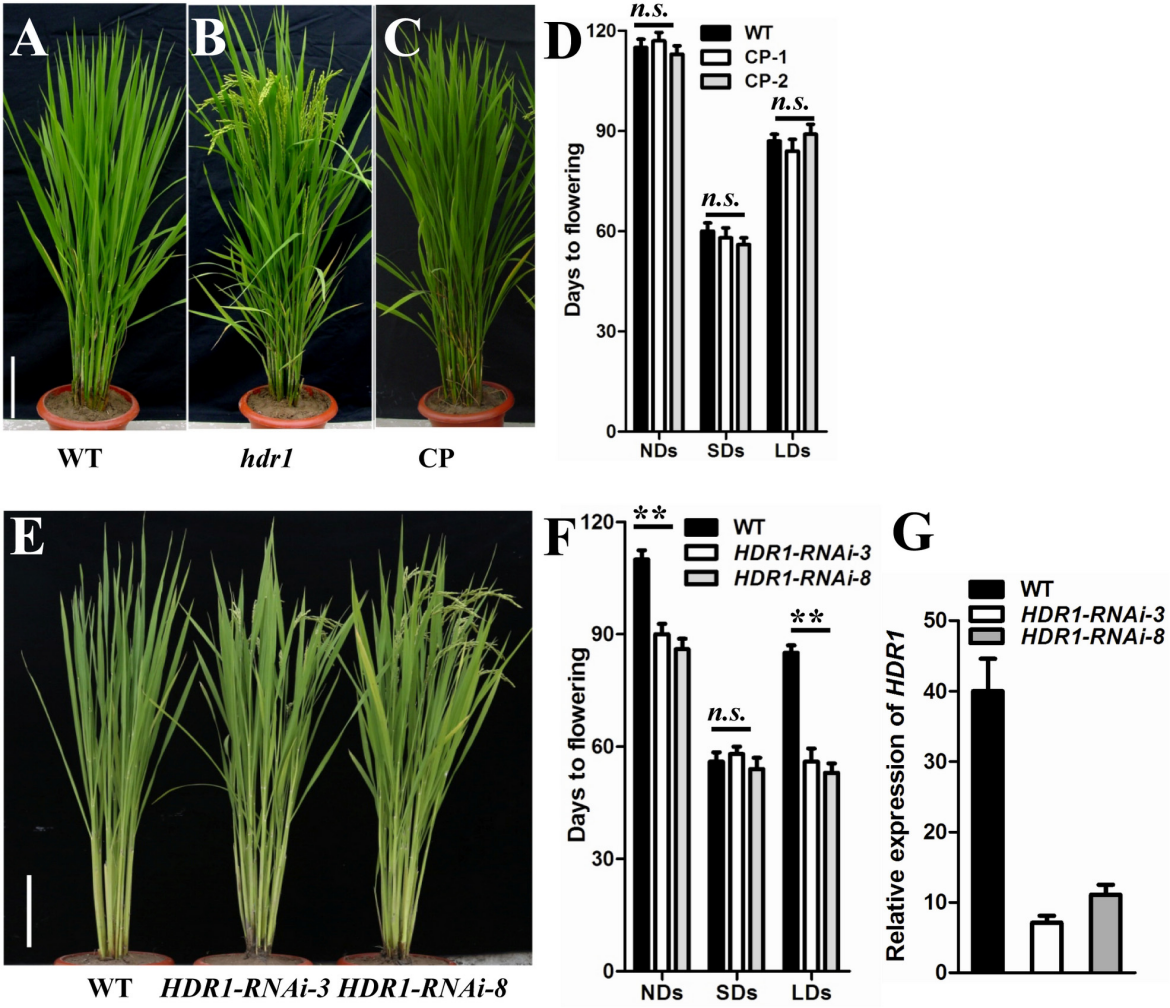
*HDR1* controls rice flowering time. (A) to (C) Complementation of *hdr1* in a paddy field. A, wild type; B, *hdr1* mutant; and C, complemented transgenic plant (CP). Bar = 20 cm. (D) Flowering time in WT and Complemented plant of transgenic lines 1 and 4 under NDs, SDs and LDs. The values presented are the mean ± s.d. (n = 12). (E) The phenotype of *HDR1*-*RNAi* plants in a paddy field. WT, left; line 3, middle; and line 8, right. (F) *HDR1*-*RNAi* plants exhibited early flowering under both NDs and LDs. The values presented are the mean ± s.d. for each of 12 independent plants of transgenic lines 3 and 8. (G) Real-time PCR analysis of *HDR1* mRNA levels in WT and RNAi seedlings; Transgenic lines 3 and 8 were used. Mean values ± s.d. were obtained from three technical replicates and two biological replicates. s.d.: standard deviations.

To verify that the early flowering phenotype we observed was caused by the disruption of *HDR1*, we performed a complementation experiment using a genomic DNA construct that included the promoter, coding, and terminator regions of *HDR1* and a full-length *HDR1* cDNA construct driven by the *HDR1* promoter. In total, 204 positive T_0_ lines were obtained and confirmed by PCR (69 lines for the genomic construct and 135 lines for the full-length cDNA construct). Of these, 142 lines (44 for the genomic construct and 98 for the cDNA construct) exhibited a flowering time of 20–28 days later than *hdr1*, similar to WT (in a paddy field in Beijing, China. From these lines, 30 complemented T_1_ plants (15 for the genomic construct and 15 for the cDNA construct) were randomly selected to produce homozygote plant for further analysis; these plants exhibited a normal flowering times compared with WT plants. Flowering time was subsequently measured in WT, *hdr1*, and complemented plants under different photoperiods. The *hdr1* plants flowered approximately 4 weeks earlier than the WT and complemented plants under LDs (14 h of light/10 h of dark) during the natural growing season (Beijing, China) (Fig 2A–2D). Slightly early flowering was also observed under SDs (Fig 2D). These data indicate that both constructs complemented the early flowering phenotype of the mutant completely (S3 Table).

To further investigate the function of *HDR1* in rice flowering time, we generated *HDR1* RNA interference (RNAi) and overexpression transgenic plants. The *HDR1-RNAi* T_0_ plants exhibited an early flowering phenotype similar to that of the *hdr1* mutant; in 118 PCR-confirmed T_0_ plants, we observed 32 lines that flowered 1 to 4 weeks earlier than WT. Furthermore, two independent T_2_ transgenic lines (*HDR1-RNAi-3* and *HDR1-RNAi-8*) exhibited early flowering under NDs and LDs, along with a dramatic reduction in *HDR1* expression levels (Fig 2E–2G). However, late flowering was not observed in paddy field-grown overexpression lines. Moreover, careful observation of plants under NDs, SDs and LDs revealed similar flowering times among the WT and transgenic overexpression plants (S1A–S1C Fig). Taken together, our data indicated that *HDR1* is a negative regulator of flowering time under LDs in rice.

### *HDR1* encodes a nuclear protein with a diurnal expression pattern

**Fig 3:**
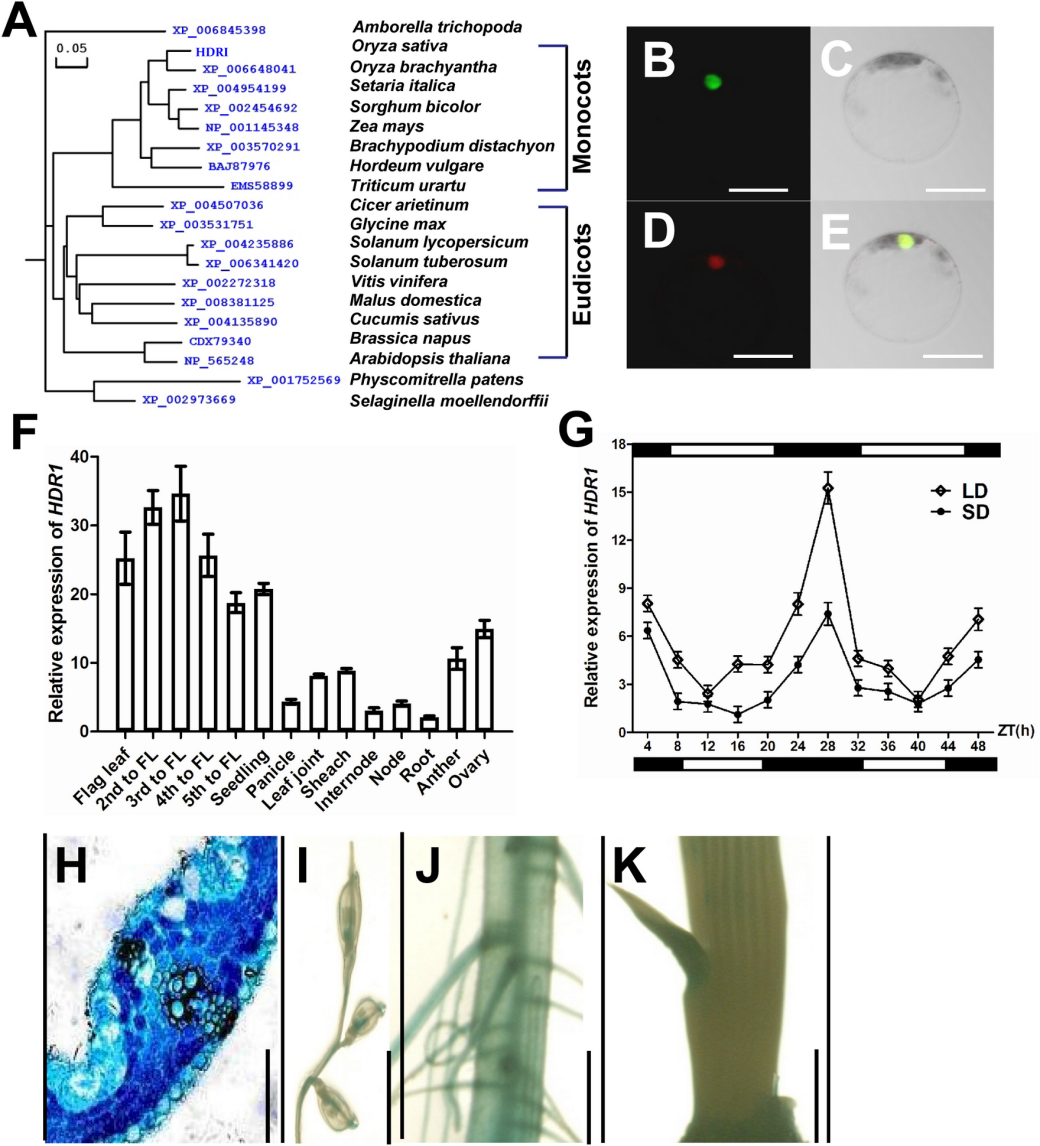
Characterization of *HDR1*. (A) Phylogeny of HDR1 homologs from different plant species. An un-rooted neighbor-joining tree was reconstructed using amino acid sequences. The length of a branch shows the relative protein sequence difference. (B) to (E) HDR1 is a nuclear protein. B, HDR1-GFP; C, Bright-field; D, OsMADS3-mCherry; and E, merged. Bar = 10 μm in (B) to (E). (F) *HDR1* transcript levels in various organs. Mean values ± s.d. were obtained from three technical replicates and two biological replicates. (G) Rhythmic expression of *HDR1*. The rice *Ubiquitin-1* (*Ubq1*) gene was used as an internal control. The values presented are the mean ± s.d. of three independent experiments and two biological replicates. The open and filled bars at the top and bottom represent the light and dark periods, respectively. s.d.: standard deviation. (H) to (K) *HDR1* expression pattern in *pHDR1*::*GUS* transgenic rice. GUS staining of a transverse section of a leaf (H), a small spikelet (I), a primary root (J), and a bud (K). Bars = 100 μm. s.d.: standard deviations.

*HDR1* in rice encodes an ortholog of PpSKI, a putative kinase ligand in moss [39,40]. HDR1-like proteins are ubiquitous in monocots, dicots, and moss, implying an ancient origin for this protein. Subsequent phylogenetic analysis revealed three corresponding main subgroups in monocots (e.g., rice, maize, sorghum, barley, millet, and wheat), eudicots (e.g., soybean, chickpea, grape, cucumber, tomato, potato, watermelon, almond tree, rape, apple tree, and *Arabidopsis*), and mosses and ferns (*Physcomitrella patens* and *Selaginellamoellendorffii*, respectively). The *Amborellatrichopoda* homolog of HDR1 formed a single clade consistent with its unique evolutionary line of flowering plants that diverged very early (~130 million years ago) from all other extant species of flowering plants (Fig 3A). We also noted that the C-terminus of the HDR1-like family is highly conserved in different species (S2 Fig).

Upon *HDR1* expression in rice leaf protoplasts, the in-frame HDR1 cDNA::GFP fusion protein was specifically localized in the nucleus (Fig 3B–3E). To investigate the expression profile of *HDR1* in rice, we examined *HDR1* expression levels in several rice tissues under LDs. Higher *HDR1* expression was detected in leaves and floral organs (Fig 3F), in accordance with the previously described expression patterns of several other flowering-time genes [19–38]. Furthermore, genomic *HDR1*fragments (including 2 kb of the promoter and part of the coding region) were fused in frame to the *β-GLUCURONIDASE* (*GUS*) gene to generate rice transgenic plants expressing the fusion protein. Histochemical staining revealed that HDR1 was strongly detected in leaves compared with other tissues (Fig 3H–3K).

Many flowering genes display a diurnal expression pattern [19–38]. We therefore examined the daily rhythmic expression pattern of *HDR1* transcripts under SDs and LDs. Under both conditions, *HDR1* expression showed a diurnal expression pattern in leaves. The transcript level began to increase after dusk, reaches a peak before dawn, and damps rapidly thereafter under both SD and LD (Fig 3G).

### *HDR1* acts as an upstream activator of *Hd1* and a repressor of *Ehd1*

**Fig 4:**
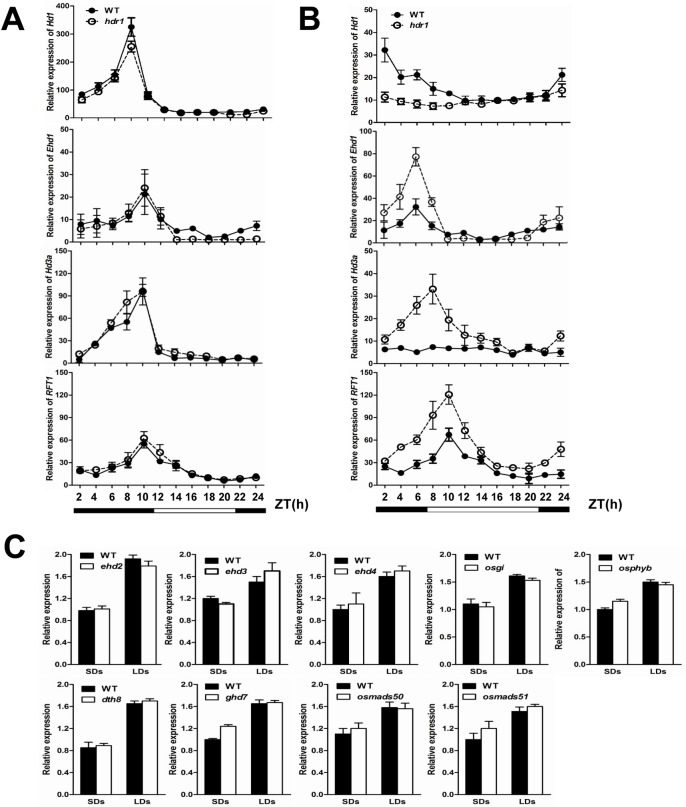
The expression patterns of *Hd1*, *Ehd1*, *Hd3a* and *RFT1* in *hdr1*, and *HDR1* in flowering-time mutants or their NILs. (A) The rhythmic expression patterns of *Hd1*, *Ehd1*, *Hd3a* and *RFT1* under SDs. The open and filled bars at the bottom represent the light and dark periods, respectively. The rice *Ubiquitin-1* (*Ubq1*) gene was used as an internal control. The values presented are the mean ± s.d. of three independent experiments and two biological replicates. (B) The rhythmic expression patterns of *Hd1*, *Ehd1*, *Hd3a* and *RFT1* under LDs. The open and filled bars at the bottom represent the light and dark periods, respectively. The rice *Ubiquitin-1* (*Ubq1*) gene was used as an internal control. The values presented are the mean ± s.d. of three independent experiments and two biological replicates. (C) Real-time PCR analysis of *HDR1* expression level in various flowering-time mutants or their NILs (near-isogenic lines) corresponding WTs. Transcriptional levels was analyzed under both SD and LD conditions. The values of folds change presented are the mean ± s.d. of three independent experiments and two biological replicates. s.d.: standard deviation.

The early flowering of *hdr1* compared with WT under LDs but not SDs is similar to the phenotypes of a previously described *Hd1* knock-down mutant [23–25] and *Ehd1*-overexpressing plants [26]. *Hd1* and *Ehd1* are two pivotal parts of the network that controls flowering in rice [13]; therefore, *Hd1* and *Ehd1* expression were studied in WT and *hdr1* plants under LDs and SDs. *Hd1* rhythmic expression was significantly decreased, whereas *Ehd1* expression was significantly increased, under LDs but not SDs in *hdr1* compared with WT plants (Fig 4A and 4B).These results suggested that *HDR1* might participates in the *Hd1* and *Ehd1* pathways to control flowering time in rice under LDs. Previous work showed that *Hd1* can repress *Ehd1* expression [44]; therefore, it is possible that the down-regulation of *Ehd1* is due to altered *Hd1* transcription.

The expression of a non-functional allele of *Hd1* or low-level expression of *Hd1* in plants causes high-level expression of the florigen gene *Hd3a*, indicating that *Hd1* represses *Hd3a* expression and delays flowering under LDs [18,25], whereas *Ehd1* increases *Hd3a* and *RFT1* expression to promote flowering under LDs [26]. To confirm whether the decreased expression of *Hd1* and increased expression of *Ehd1* in *hdr1* are responsible for the up-regulation of *Hd3a* and *RFT1* and the subsequent promotion of flowering in rice, we examined the rhythmic expression patterns of *Hd3a* and *RFT1* in mutant and WT plants. Under SDs, *Hd3a* and *RFT1* expression remained unchanged between *hdr1* and WT (Fig 4A). However, *Hd3a* and *RFT1* expression increased significantly compared with the levels in WT in the light and were maintained at low levels in the dark under LDs (Fig 4B). These results were consistent with the conclusion that the two *FT-like* genes *Hd3a* and *RFT* ensure flowering in rice under LDs [13,22].

*HDR1* rhythmic expression in *hd1*-mutant and *ehd1* plants was also analyzed. *HDR1* expression did not differ significantly in *hd1*-mutant plants compared with WT under LDs (S3A Fig). The lack of change in *HDR1* expression in the *hd1* background and the significant suppression of *Hd1* expression in an *hdr1* background under LDs demonstrates that *HDR1* is an upstream regulator of *Hd1*. *HDR1* expression was not also significantly affected in *ehd1* plants, indicating that *Ehd1* might encode a downstream component of *HDR1* (S3A Fig).

*OsGI* [18,19,23–25], *OsPhyB* [45], *Ehd2* [28–30], *Ehd3* [37], *Ehd4* [38], *Ghd7* [34], *DTH8* [35], *OsMADS50* [31], and *OsMADS51* [27] are important genes under the control of *Hd1* and *Ehd1* expression under LDs. We next investigated whether these regulatory genes are down- or up-regulated by *HDR1*. The expression levels of *OsGI*, *OsphyB*, *Ehd2*, *Ehd3*, *Ehd4*, *Ghd7*, *DTH8*,*OsMADS50*, and *OsMADS51* in the *hdr1* background were similar to WT under LDs (S3C Fig). When we tested the expression levels of *HDR1* in *osgi*, *osphyb*, *ghd7*, *dth8*, *osmads50*, *osmads51*, *ehd2*, *ehd3* and *ehd4* mutants or nonfunctional NIL, the qPCR assay did not indicate down or up-regulation of these flowering-time regulation genes (Fig 4B). The expression data indicated that *HDR1* function as flowering regulator, independent of *OsGI*, *OsphyB*, *hd2*, *Ehd3*, *Ehd4*, *Ghd7*, *DTH8*, *OsMADS50*, and *OsMADS51*. In addition, a yeast two-hybrid assay did not indicate any direct association between HDR1 and these flowering regulators to regulate flowering in rice (S4B Fig). We further carried out a yeast one-hybrid assay to investigate whether HDR1 can bind the promoter regions of *Hd1* and *Ehd1*. However, HDR1 could not bind the promoter regions of *Hd1* and *Ehd1* (S8 Fig).

### HDR1 and OsK4 proteins form nuclear complexes

**Fig 5:**
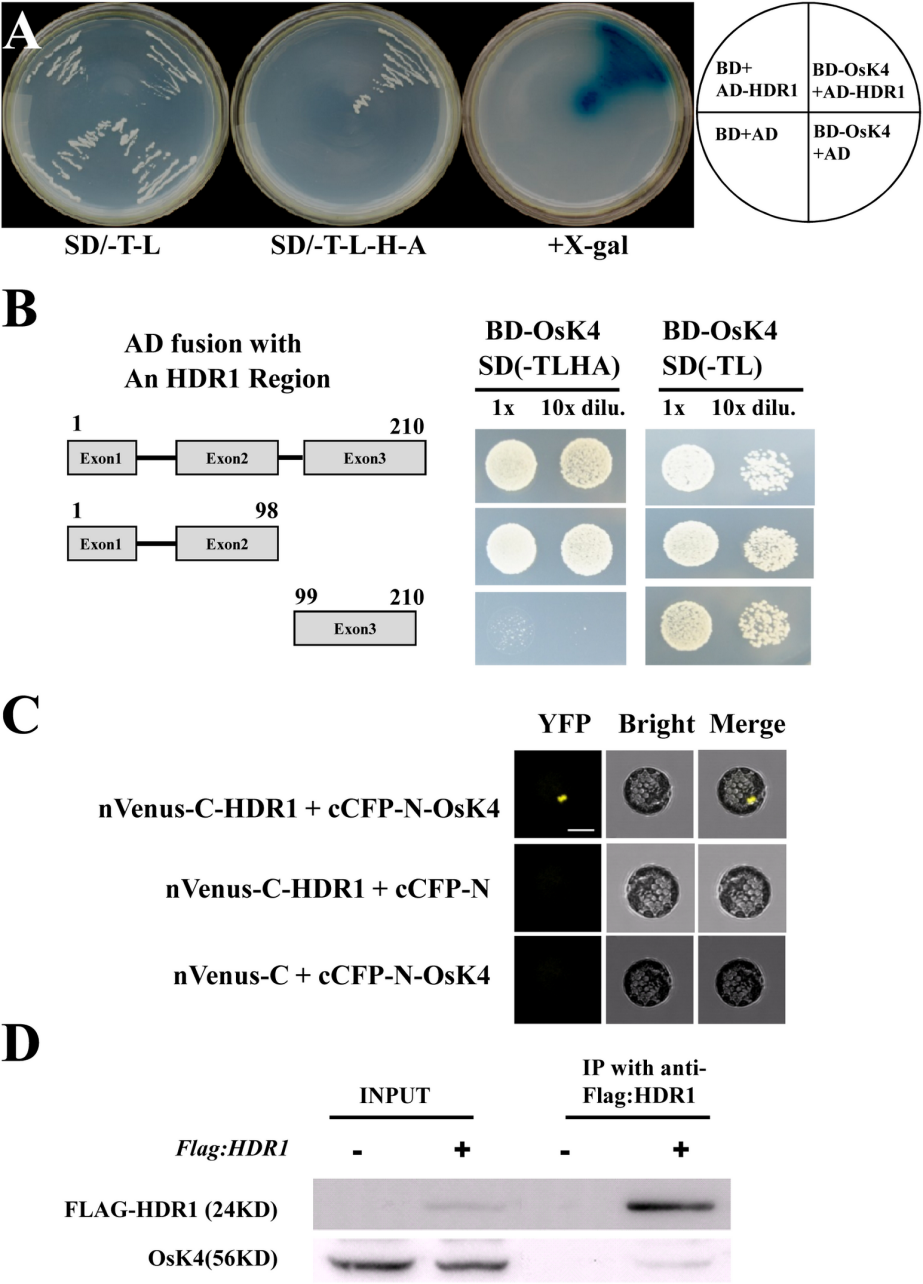
HDR1 interacts with OsK4. (A) HDR1 directly interacted with kinase OsK4 in the Y2H system. (B) Exon1 and Exon2 of HDR1, but not Exon3, directly interacted with OsK4 in yeast cells. (C) BiFC analyses of the interactions of HDR1 with OsK4 in *Arabidopsis* protoplast. YFP signals resulted from the physical association of HDR1 with OsK4 proteins, not negative pairs, in the nuclei. Scale bar = 10μm. (D) Co-immunoprecipitation of HDR1 with OsK4 in rice plant. Total protein extracted from the leaves expressing FLAG-HDR1 and WT, and then FLAG-HDR1 was immunoprecipitated with anti-FLAG, followed by western blotting to analyze the OsK4 with OsK4 antibody.

*HDR1* encodes a homolog of the ligand of the SNF1/AMPK/SnRK1 kinase PpSKI in moss [40]. Several kinase genes belonging to the SnRK family have been identified in plants, including *Arabidopsis* and rice [46–49]. A yeast two-hybrid (Y2H) library, which consisted of different developmental stages leaf and floral tissues, was screened to identify interacting kinase of HDR1 in rice. Using HDR1 as bait, multiple independent fragments for many interacting proteins were identified. Among them, 5 independent clones that fulfilled the criteria of interaction with HDR1 were isolated and sequenced. The different sequences of these 5 clones match to be a gene *OsK4* (LOC_Os08 g37800 [TIGR]). A protein with 97.84% similarity to OsK4, named OsK3 (Os03 g0289100 [TIGR]), also exists in the rice genome (S5A Fig). Firstly, we conducted yeast two-hybrid assays to confirm direct interaction of OsK4 and OsK3 with HDR1. Indeed, OsK4 and OsK3 both directly interacted with HDR1 (Fig 5A, S5B Fig). Detailed assays were also performed to clarify the role of the different exons of HDR1 in interacting with OsK4 in the Y2H system, which revealed that exon1 and exon2, but not exon3, strongly interacted with OsK4. Given the evolutionary conservation of the C-terminus of HDR1, these data indicated that the N-terminus of HDR1 is important for interacting with OsK4 (Fig 5B and S2 Fig). In addition, we also noted that *hdr1* exhibited late germination, similar to that reported for *osk3* and *osk4* mutants (S6 Fig and [48]).

To investigate the subcellular localization of the HDR1 and OsK4 interaction *in vivo*, bimolecular fluorescence complementation (BiFC) was employed to confirm direct interaction between HDR1 and OsK4 using *Arabidopsis* protoplasts. As expected, YFP fluorescence was only detected in *Arabidopsis* protoplasts co-transfected with pSAT1-nVenus-C-HDR1 and pSAT1-cCFP-N-OsK4, not two negative controls of pSAT1-nVenus-C-HDR1/pSAT1-cCFP-N and pSAT1-nVenus-C/pSAT1-cCFP-N-OsK4, confirming the physical interaction of HDR1 and OsK4 *in vivo* (Fig 5C). Next, we conducted co-immunoprecipitation (Co-IP) experiments to verify the interaction between HDR1 and OsK4. For this purpose, we generated transgenic lines expressing *FLAG-HDR1* driven by *Actin1* promoter could rescue *hdr1* phenotype (S10 Fig), and utilized an OsK4 antibody that could specifically recognize the OsK4 protein (S7 Fig). We found that anti-FLAG (recognizing FLAG-HDR1) could efficiently immunoprecipitate OsK4, revealing that OsK4 associated with HDR1 (Fig 5F). Collectively, these data revealed that HDR1 strongly interacts with OSK4.

### *OsK4* acts similarly to *HDR1* to repress flowering

**Fig 6:**
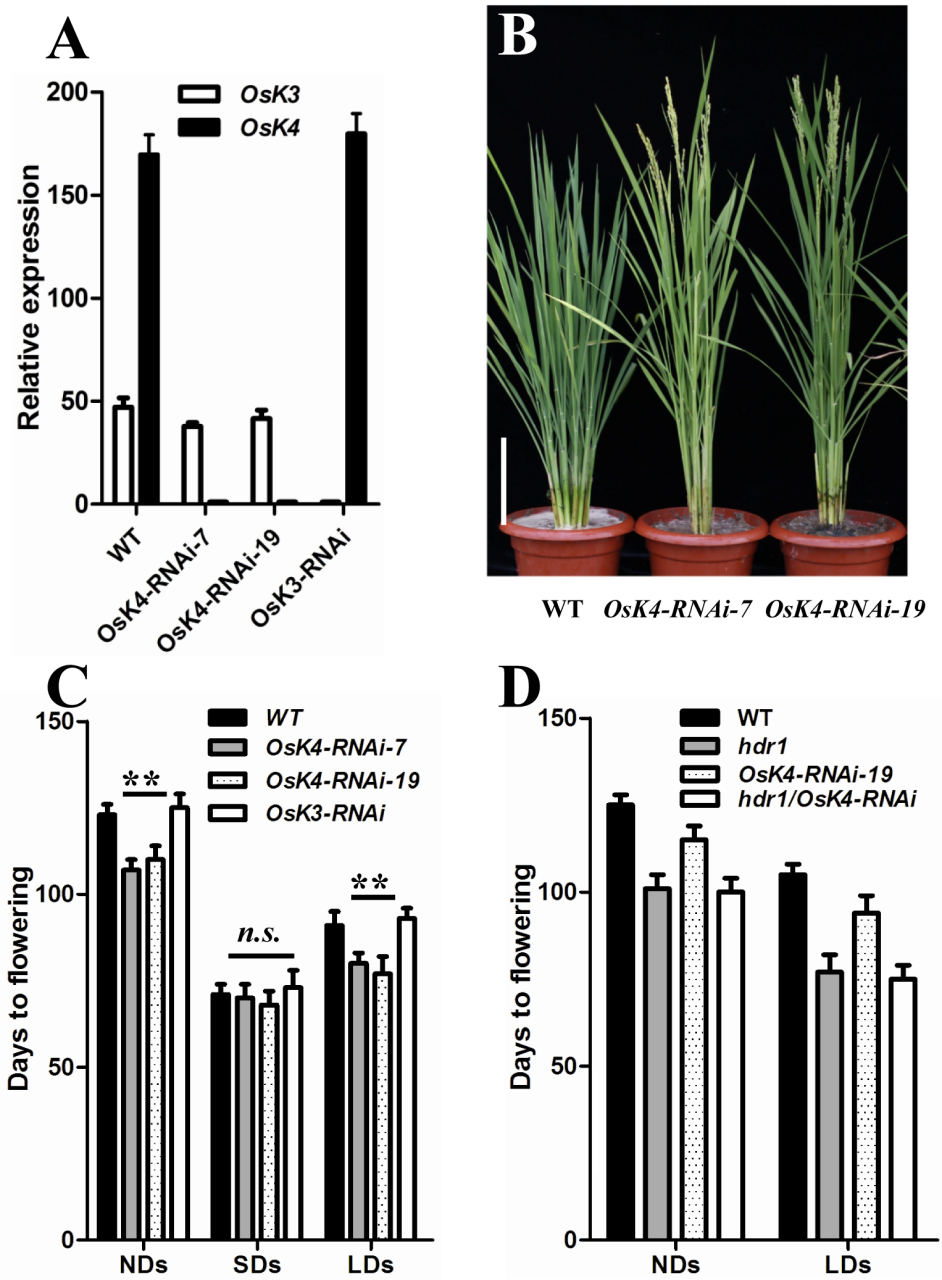
*OsK4-RNAi* plants exhibit early flowering. (A) The mRNA expression levels of *OsK3* and *OsK4* in WT and RNAi plants. The values presented are the mean ± s.d. of three independent experiments and two biological replicates. (B) The early flowering phenotype of *OsK4*-RNAi plants in a paddy field (middle and right). WT Nipponbare (left). (C) Flowering time of *OsK3-RNAi*, *OsK4-RNAi* and WT plants under different day-length conditions (n = 12). NDs, natural day conditions; SDs, short day conditions; LDs, long day conditions. (D) Flowering time of *hdr1*, *OsK4-RNAi*, *hdr1*, *OsK4-RNAi* and WT plants under NDs and LDs (n = 12). s.d.: standard deviations.

To investigate the function of *OsK4* in rice flowering, we generated *OsK4*- and *OsK3*-*RNAi* plants. The RNAi lines were first identified using a GUS activity assay (the vector pTCK309 contains a *β-GLUCURONIDASE* (*GUS*) gene derived by the 35S promoter within the T-border, which enables the convenient identification of transgenic plants), and then confirmed by qPCR (Fig 6A). The *OsK4*-*RNAi* lines flowered approximately 2 weeks earlier than WT, whereas no early flowering plants were observed among the *OsK3-RNAi* lines (Fig 6B and 6C). We further constructed the *hdr1 OsK4-RNAi* double mutant, and found it flowered similarly with *hdr1* mutant in LDs (Fig 6D).

**Fig 7:**
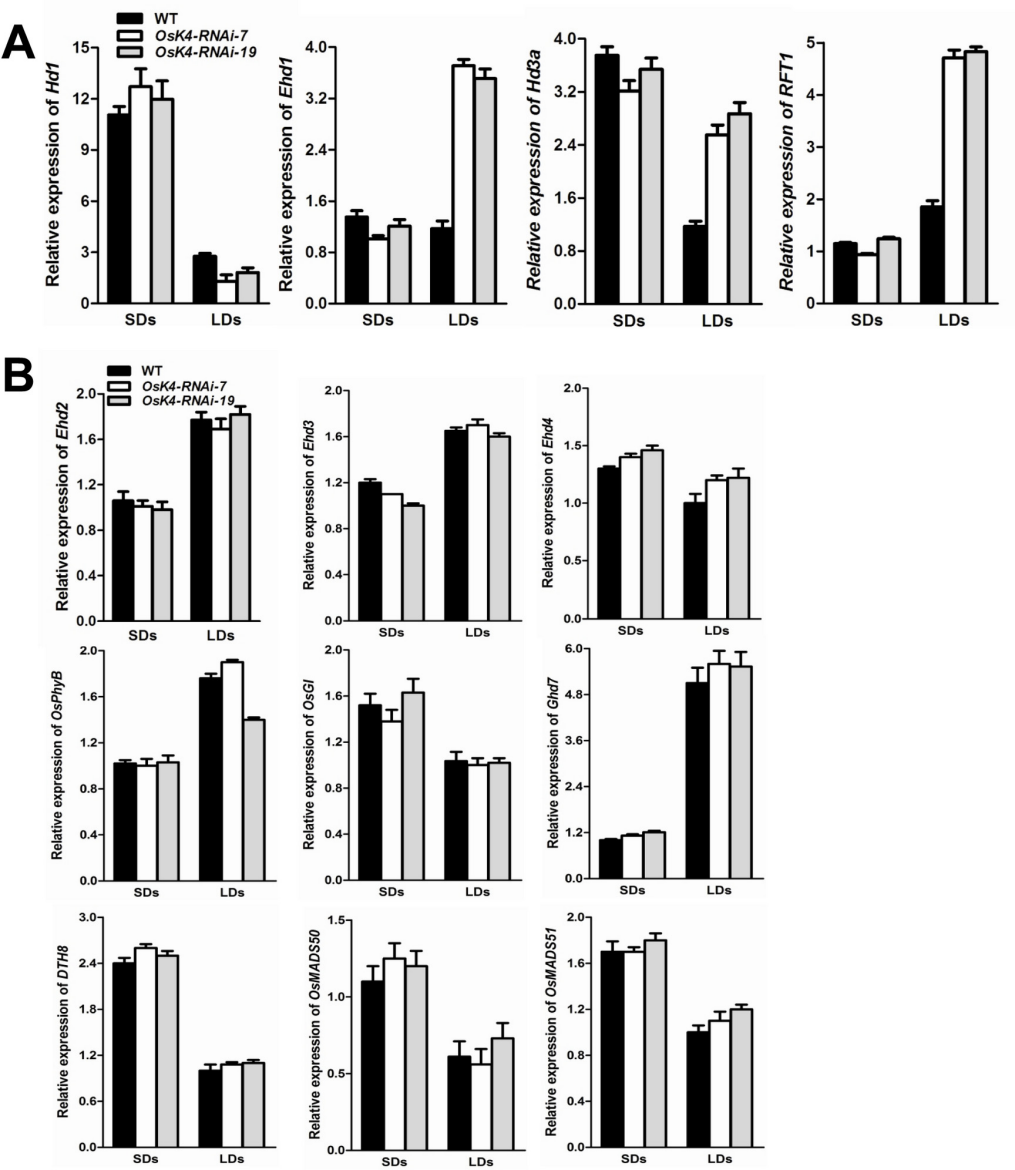
The expression patterns of flowering genes in *OsK4*-*RNAi* plants are similar to those in *hrd1*. (A) The expression patterns of *Hd1*, *Ehd1*, *Hd3a* and *RFT1* under SDs and LDs. The rice *Ubiquitin-1* (*Ubq1*) gene was used as an internal control. The values presented are the mean ± s.d. of three independent experiments and two biological replicates. (B) Real-time PCR analysis of representative flowering-related genes in WT and *OsK4-RNAi* plants. Nine reported flowering-related genes (*Ehd2*, *Ehd3*, *Ehd4*, *OsGI*, *OsPhyB*, *OsMADS50*, *OsMADS51*, *Ghd7* and *DTH8*) were analyzed under both SDs and LDs. The values presented are the mean ± s.d. of three independent experiments and two biological replicates. s.d.: standard deviations.

The early flowering of the *hdr1* and *OsK4*-*RNAi* plants coupled with the interaction between HDR1 and OsK4 raised the question of whether a similar regulatory pathway involving *HDR1* and *OsK4* operates in rice under LD. qPCR analyses were conducted under LDs and SDs, but the upregulation of *Ehd1* and downregulation of *Hd1* were detected only under LD, confirming that *OsK4* exhibited the same pattern of regulation as *HDR1*. Similarly, the increase in *Ehd1* and steep decrease in *Hd1* caused the upregulation of the florigens*RFT1* and *Hd3a* was observed only under LD (Fig 7A).

*OsGI*, *OsPhyB*, *Ehd2*, *Ehd3*, *Ehd4*, *Ghd7*, *DTH8*, *OsMADS50* and *OsMADS51* were also investigated in *OsK4-RNAi* plants to check for an alternative regulatory pathway. The two RNAi lines exhibited no obvious differences in expression compared with WT plants (Fig 7B).

### OsK4 associated with HDR1 functions to phosphorylate HD1

**Fig 8:**
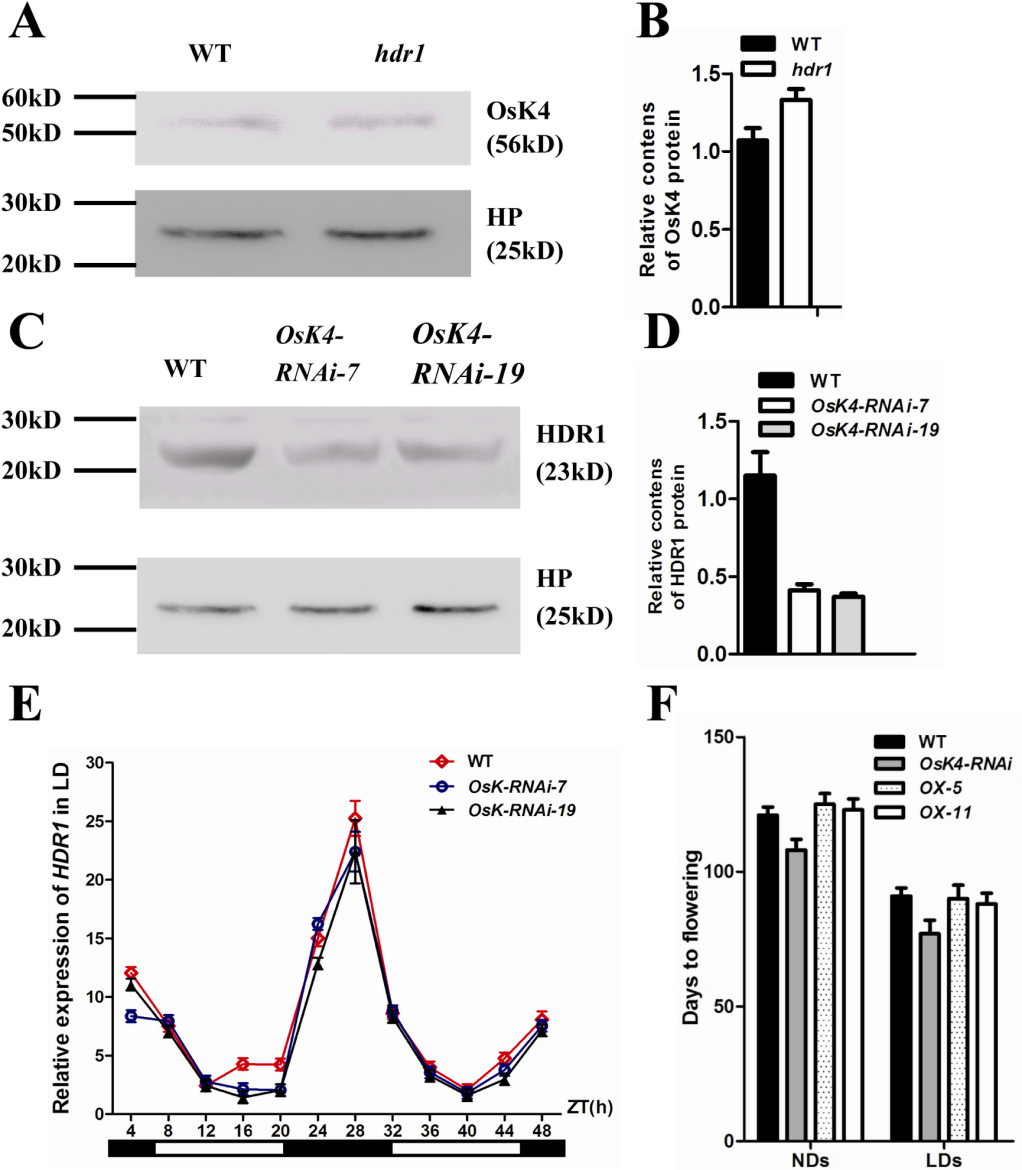
The relationship between HDR1 and OsK4. (A) OsK4 protein level remains unchanged in *hdr1*. The Heat-shock Protein (HP) was used as a loading control. Total protein extracted from the 40 d old seedlings of WT and *hdr1*, was subjected to precipitates with anti-OsK4 or anti-HP respectively by western blotting analysis. (B) OsK4 protein intensity in WT and *hdr1*, quantified by ImageJ program. (C) HDR1 protein level is decreased in *OsK4*-*RNAi* transgenic lines 7 and 19. The HP was used as a loading control. Total protein extracted from the 40 d old seedlings of WT, *OsK4-RNAi* transgenic lines 7 and 19, were subjected to precipitates with anti-HDR1 or anti-HP respectively by western blotting analysis. (D) HDR1 protein intensity in WT and *OsK4-RNAi* transgenic lines 7 and 19, quantified using ImageJ program. (E) The expression rhythm of *HDR1* in *OsK4-RNAi-7* and *OsK4*-*RNAi-19* plants. (F) Overexpression of *HDR1* rescused the phenotype of *OsK4*-*RNAi*.

To clarify the relationship between HDR1 and OsK4, we first analyzed the localization and expression of OsK4 and HDR1 in *hdr1* or *OsK4*-*RNAi* transgenic plants. In *hdr1*, the OsK4-GFP fusion protein localized to the nucleus (S9A–S9D Fig), in accordance with a previous report [48]. To investigate whether a lack of HDR1 function affects the OsK4 protein level, we performed western blotting to measure OsK4 protein levels in *hdr1* and WT, and found no differences in the levels of OsK4 (Fig 8A and 8B). Two transgenic lines were used to determine the HDR1-GFP fusion protein location. HDR1 was exclusively localized to the nucleus, as in WT (S9E–S9L Fig). Interestingly, HDR1 protein levels were dramatically decreased in *OsK4*-*RNAi* plants (Fig 8C and 8D).

The low HDR1 levels in *Osk4*-*RNAi* lines may due to the low *HDR1*; therefore, we examined the *HDR1* gene rhythmic expression pattern in WT and the *OsK4-RNAi* lines. The expression rhythm of *HDR1* was not changed in *OsK4-RNAi* (Fig 8E). We also found that overexpression of *HDR1* could rescue the early flowering phenotype of OsK4*-RNAi* plants (Fig 8F).

**Fig 9:**
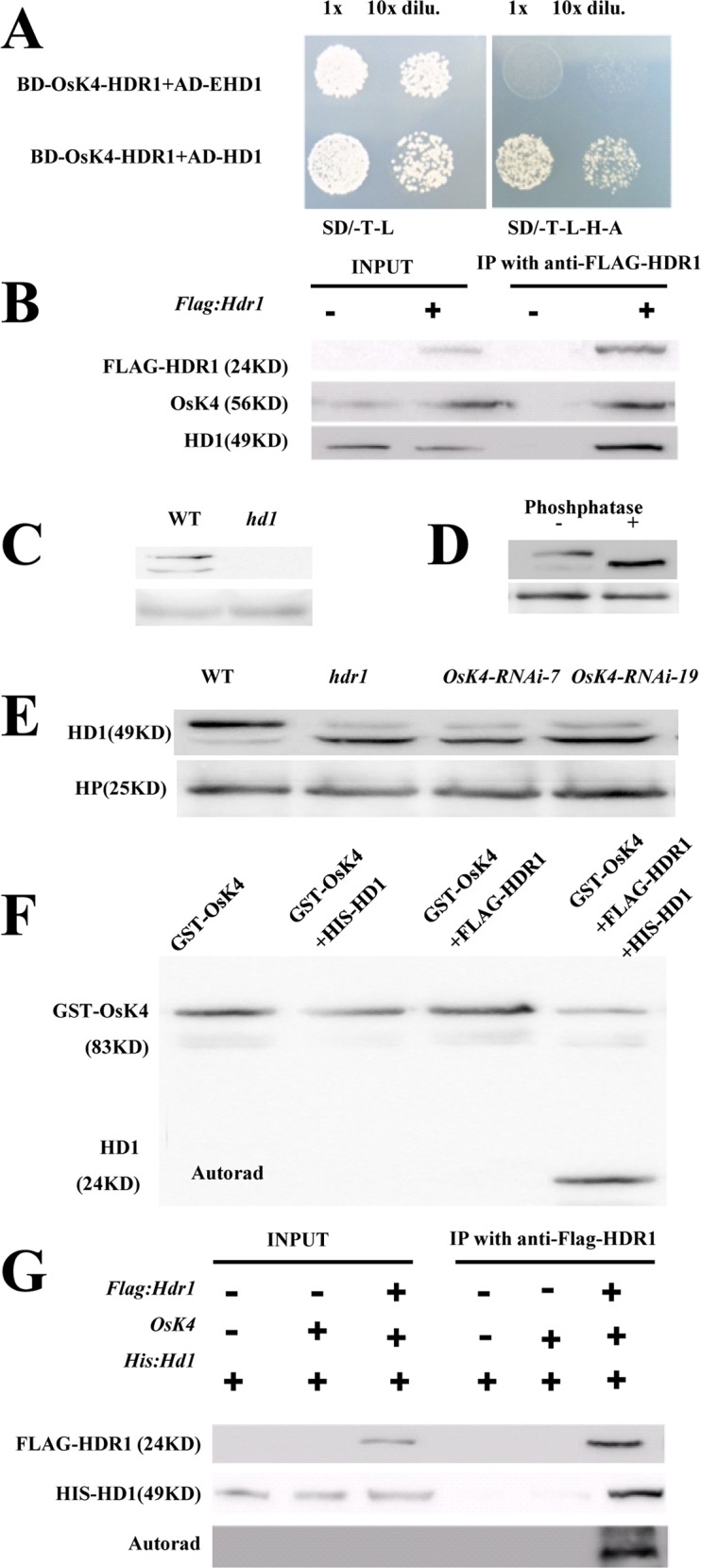
OsK4 associated with HDR1 function to phosphorylate HD1. (A). HDR1-OsK4 directly interacted with HD1 in the Y3H system. (B). Co-immunoprecipitation of HDR1-OsK4 with HD1 in rice plant. Total protein extracted from the leaves expressing FLAG-HDR1 and WT, then, FLAG-HDR1 was immunoprecipitated with anti-FLAG, followed by western blotting to analyze the HD1 and OsK4 with specific antibody. (C) Two different forms of HD1 were detected by anti-HD1 antibody in WT.HP protein was used as a loading control. Proteins isolated form 40 d seedlings grown under LDs (14 h light/10 h dark). (D) The slower-migrating form of HD1was susceptible to phosphatase treatment. Nuclear proteins were treated with lambda (λ) phosphatase. Anti-HD1 antibodies were used to detect HD1 protein and HP as a loading control. (E) The different forms of HD1 in WT, *hdr1* and *Osk4-RNAi* plants. The main forms of HD1 are phosphorylated in WT, but in *hdr1* and *Osk4-RNAi* were non-phosphorylated. HP protein was used as a loading control. Proteins isolated form 40 days seedlings grown under LDs (14 h light/10 h dark). (F) *In vitro* labeling test of HD1 protein with radioactive phosphate. The recombinant purified proteins of *OsK4*, *Hd1* and *HDR1* were incubated with radioactive phosphate. (G) OsK4 depended on HDR1 to phosphorylate HD1 *in vivo*. OsK4-HDR1 complex was immunoprecipitated from transgenic *Flag*:*HDR1* plants. Anti-HD1 antibody was used to detect HD1 protein and HP as a loading control.

Given our findings that HDR1 and OsK4 did not directly interact with HD1 or EHD1 (S4A Fig), but that HDR1 acted as an upstream regulators of *Hd1* and *Ehd1* and could form a complex with OsK4, we explored the possibility of a physical interaction between HDR1-OsK4 and HD1 or EHD1. We performed Y3H assays with HD1 or EHD1 fused to the GAL4 activation domain (AD), and OsK4 fused to the GAL4-binding domain (BD) along with HDR1 co-expression. We found that, indeed, HDR1 and OsK4 together could interact with HD1, not EHD1 (Fig 9A). To further verify that HDR1 and OsK4 interacted to HD1, a Co-IP assay was carried out. We found that HDR1 and OsK4 effectively precipitated HD1 when FLAG-HDR1 and OsK4 were input together, indicating that HD1 is part of the HDR1-OsK4 complex (Fig 9B).

It was reported that the HD1 ortholog CO in *Arabidopsis* could be phosphorylated [50]. We found accordingly that two forms of HD1 protein migrated differently after electrophoresis in WT, but none HD1 protein was detected in *hd1* (Fig 9C). To examine whether phosphorylation of HD1 contributes to these different forms, nuclear protein extracts of WT were incubated with λ phosphatase. After incubation of protein phosphatase, the slower-migrating form of HD1 was no longer detected (Fig 9D). The direct interaction of HDR1 and OsK4 with HD1 *in vivo* raise the question of whether OsK4 phosphorylates HD1, as well as the role of HDR1 in the process of HD1 phosphorylation. Firstly, we performed western blot to verify phosphorylated HD1 level in WT, *hdr1* and *OsK4*-*RNAi* plants. Two forms of HD1 protein migrated differently after electrophoresis in WT and mutant lines, and less phosphorylated HD1 protein was detected in *hd1* and *OsK4-RNAi* lines (Fig 9E). To verify the phosphorylation of the HD1, we carried out an *in vitro* phosphorylation assays. Using the recombinant purified proteins GST-OsK4, FLAG-HDR1 and HIS-HD1 incubated with [γ-32P] ATP, autoradiogram indicated only in the presence of HDR1, OsK4 could phosphorylate HD1 (Fig 9F). We further confirmed that OsK4 immunoprecipitated from *Flag*:*HDR1* plants could phosphorylate HD1 *in vivo* (Fig 9G). All the data gave clues that HDR1was necessary for the phosphorylation of HD1 by OsK4.

## Discussion

In this study, we have identified HDR1 interacted with OsK4 kinase protein, and was essential for inhibiting flowering by regulating the expression of *Hd1* and *Ehd1*, leading to the downregulation of *Hd3a* and *RFT1* under LD. We have further determined that OsK4 and HDR1 could phosphorylate HD1. These findings collectively illustrated the important role of the HDR1 and OsK4 complex in the day-length regulation of flowering time via HD1.

### *HDR1* transcriptionally regulates *Hd1* to control flowering time in rice

Rice is known as a short-day plant, and most rice cultivars may be induced to flower more rapidly under SDs than LDs. There are at least two independent flowering pathways, *Hd1* and *Ehd1*, that regulate floral transition in rice [13,21]. The *Hd1* pathway is conserved between rice and *Arabidopsis*, but the *Ehd1* pathway is unique to rice [21]. Our results reveal that *HDR1* shows a similar diurnal expression pattern with *Hd1* and *Ehd1*, which accumulates after dusk, reaches a peak before dawn, and damps rapidly thereafter under both SD and LD (Fig 3G). The expression pattern implicated *HDR1* in photoperiodic control of flowering through the regulation of *Hd1* and *Ehd1*. Upon the loss of HDR1 function, *Hd1* rhythmic expression was significantly decreased under LDs but not SDs, whereas *Ehd1* expression was significantly increased (Fig 4A and 4B). Previous studies have indicated that *Hd1* may function to repress *Ehd1* expression [44]. The results of the present study indicated that *Hd1* expression is suppressed, and *Ehd1* expression is promoted by *HDR1* under LDs (Fig 4B). Thus, *HDR1* might integrate different pathways in the regulation of flowering, in which *Hd1* is upregulated by *HDR1*to repress *Ehd1* expression, and may also be independently involved in the *Ehd1* pathway.

**Fig 10:**
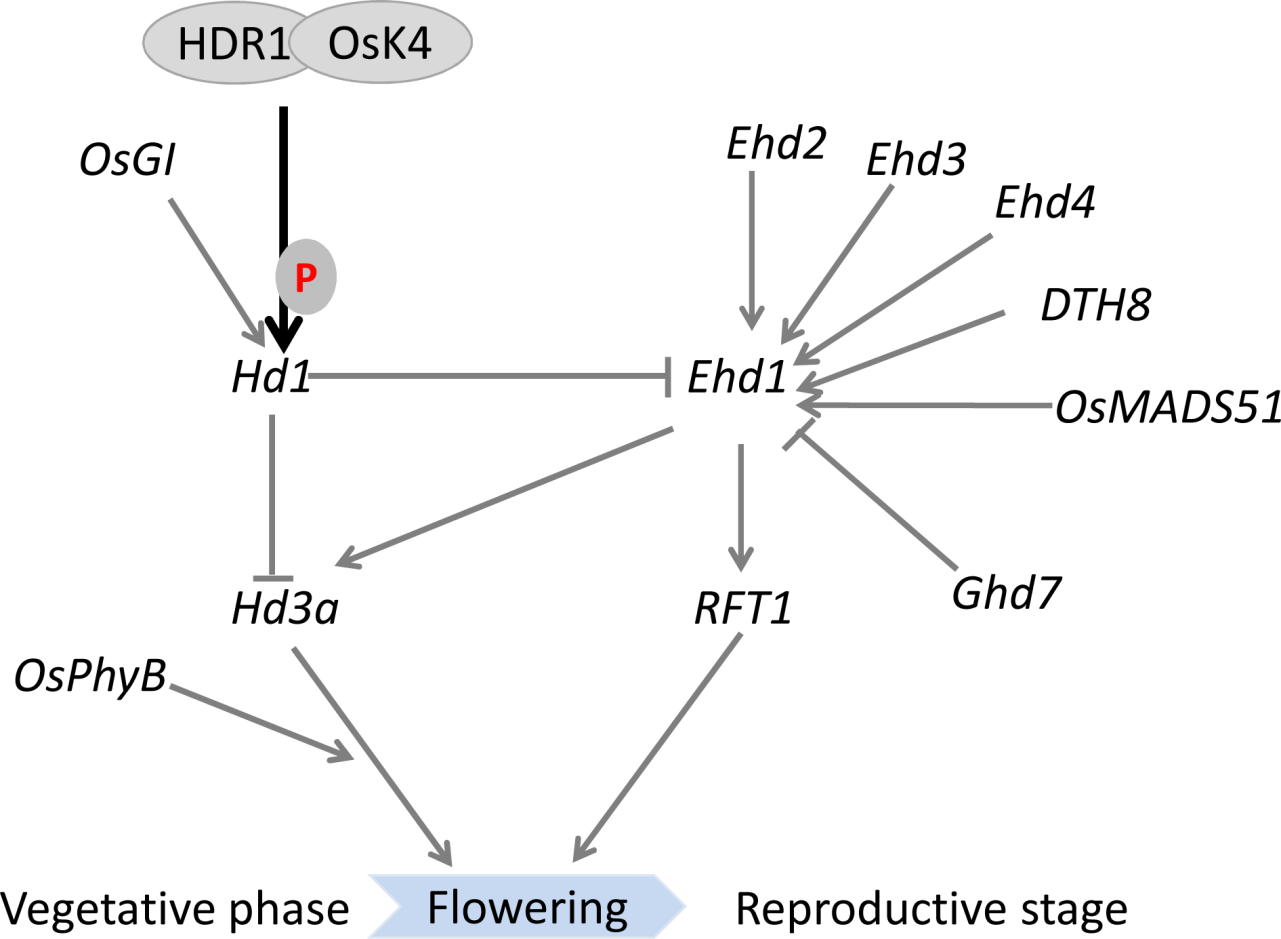
A working model of *HDR1*- and *OsK4*-mediated flowering pathway in rice. Rice flowering is mainly regulated by the *Hd1*-dependent and *Ehd1*-dependent pathways. HDR1 and OsK4 come together to regulate *Hd1* and *Ehd1* expression and phosphorylated HD1 in the control of flowering time in rice. The black lines show regulators that have added to this flowering network from our study, and the gray lines indicate previously identified genes. P: indicates Phosphorylation.

Several genes that control the *Hd1* and *Ehd1* pathway have been identified and studied, including *OsGI*, *Ehd2*, *Ehd3*, *Ehd4*, *Ghd7*, *DTH8*, *OsMADS50* and *OsMADS51* [27–38]. In an effort to explore the relationship between HDR1 and these known regulators, we first examined whether the expression levels of these genes were altered in the relevant mutants. The expression levels of *HDR1* and these genes remain unchanged in WT and mutants under LD (Fig 4C and S3C Fig). We also observed the nuclear localization of HDR1 which suggests this protein may function to form nuclear complexes with regulatory factors to control flowering time. However, *HDR1* did not associate with these flowering regulators (S4B Fig). These results suggested that *HDR1* is a novel regulator of flowering time, independent of *OsGI*, *Ehd2*, *Ehd3*, *Ehd4*, *Ghd7*, *DTH8*, *OsMADS50*, and *OsMADS51* (Fig 10). However, *HDR1* was unable to bind the *Hd1* and *Ehd1* promoters (S8 Fig), indicating that there are other components to media the binding of this complex or *Hd1* and *Ehd1* are likely indirect targets.

### HDR1 is associated with OsK4 to control flowering in rice

*HDR1* is ubiquitous in plants, including mosses, implying its ancient origin. The only reported homolog is *PpSKI* [40], which encodes a ligand of the kinase *SnRK1* in moss. The deletion of *PpSKI* increases gametophore formation and reduces protonemal growth under low-light conditions but not under normal light conditions in moss [40]. However, *HDR1* functions in floral transition in rice, and disruption of *HDR1* shows early flowering. Moreover, the PpSKI-interacting protein SnRK1, which is a Snf1-related protein kinase found in yeast, animals, and plants, is required for necessary metabolic changes in plants during dark hours [39]. In plants, Snf1-related kinases participate in the regulation of many physiological processes, including the cell cycle, meristem development, and pathogen responses [49]. As predicted, HDR1 act as a Snf1-related kinase interactor 2, and associated protein OsK4, a Snf1-related kinase has been identified [39].We postulated that this complex may function to phosphorylate target proteins. To test this hypothesis, we first analyzed the localization and expression of *OsK4* and *HDR1* in *hdr1* or *OsK4-RNAi* transgenic plants to clarify the relationship between *HDR1* and *OsK4*. In *hdr1*, the OsK4-GFP fusion protein localized to the nucleus (S9A–S9D Fig), in accordance with a previous report [48]. Interestingly, the HDR1 protein levels were dramatically decreased in *OsK4-RNAi* plants (S9E–S9L Fig). The fact that disrupting *OsK4* function leads to effect steady-state levels of *HDR1* expression suggests that *OsK4* functions to affect the protein levels of HDR1. These results collectively indicated that the early flowering phenotype in *OsK4-RNAi* plants might be regulated by a decrease in HDR1 protein levels.

Our study indicated that HDR1 and OsK4 regulate *Hd1* expression at transcriptional level. Protein interaction analyses support that HDR1-OsK4 directly interacts with HD1 at protein level. These data suggested that might exist a feedback way to regulate *Hd1*, The likely regulating model could be found in some important functional gene in plants. Auxin response factors (ARFs), NPH4/ARF7 and ARF19, could bind auxin response promoter elements and mediate gene transcription [51]. Auxin induces the *ARF19* gene, and NPH4/ARF7 and ARF19 together are required for its expression, suggesting NPH4/ARF7 and ARF19 may activate *ARF19* in a positive feedback loop [51]. *Hd1* like *ARF19*, as above described, is regulated in a positive feedback way needed HDR1-OsK4 functionally.

### OsK4 functions depend on HDR1 to phosphorylate HD1

As mentioned above, OsK4 and HDR1 act as a Snf1-related kinase and a Snf1-related kinase interactor 2, respectively. Recently studies indicated that the protein kinase *CK2* (*Hd6*) might control *Hd1* activity through the phosphorylation of an unknown interacting factor to control flowering time in rice [50]. We thought that HDR1-OsK4 might also phosphorylate HD1, although the expression level of *Hd1* is regulated by HDR1 and OsK4. As expected, HDR1-OsK4 could form nuclear complexes with HD1 *in vivo*, and phosphorylate HD1 (Fig 9C). The HDR1 is key component of the complex to assist phosphorylation of HD1. Taken together, these findings suggest that HDR1-OsK4 acts to downregulate *Hd1/Ehd1* expression and phosphorylate Hd1 to prevent early flowering in LDs, conferring a photoperiodic response.

AtSnRK1, the OsK4 ortholog in *Arabidopsis*, is an atypical AMPK. AtSnRK1 family members comprise a catalytic α subunit and non-catalytic β and γ subunits, and multiple isoforms of each subunit type exist, giving rise to various isoenzymes [52]. This suggested that HDR1 might functionally act as non-catalytic β or γ subunits in rice. It is reported that the SnRK1 kinase complex are necessary to phosphorylate regulated transcription factors [46,49]. Hence, the phosphorylation of HD1 needs not only catalytic subunit OsK4, but also subunits such as HDR1 presenting. It has not reported that phosphorylated transcription factors prior to combine the kinase complex or not [46,49], whether HDR1 can only interact with phosphorylated Hd1 needed further illuminated later.

## Materials and Methods

### Plant materials and measurements

The *Tos17*-tagged mutant *hdr1* was identified from our T-DNA insertion rice mutant library (*O*. *sativa* L. spp. *japonica* cv. Nipponbare) [41–43]. Screening for flowering time mutants was carried out by planting the mutant materials under natural LD conditions in the experimental field at the Chinese Academy of Agricultural Sciences in Beijing (39°54'N, 116°23'E), China, from May to October of each year. The agricultural traits of mutant and WT plants were recorded in three growing seasons.

Rice plants were also grown in a growth chamber under LD (14 h light/10 h dark) and SD (10 h light/14 h dark) with a light intensity of 800 μmol•m^-2^•s^-1^. To analyze the diurnal expression patterns of flowering genes and flowering time, the materials were first grown under NLD for 30–40 days and then transferred to LD or SD for 10 days.

### *hdr1* mutant isolation and complementation

Genomic DNA flanking the *Tos17* left border was cloned using PCR-based genome walking with nested specific primers according to Peng, with modifications [42]. The modified primers were TB1, TB2, AP1 and AP2. The products were sequenced using TB2 as the sequencing primer. The *Tos17* insertion site in *HDR1* was identified by BLAST searches of the rice genome (http://blast.ncbi.nlm.nih.gov/Blast.cgi) using the rescued flanking sequences.

Two constructs were used for complementation testing. For the cDNA construct, the full-length *HDR1* cDNA (amplified using primer pair HDR1-cp3-F and HDR1-cp3-R, S1 Table) was cloned into the binary vector pCam2300K, which was modified from pCambia2300Actin1 carrying the 1.8-kb *HDR1* promoter (amplified using primer pair HDR1-cp1-F and HDR1-cp2-R, S1 Table), to generate *pHDR1*::*HDR1*. For the genomic construct, a 5.1-kb fragment containing the promoter, coding, and terminator regions of *HDR1* was amplified from genomic DNA (using the primer pair HDR1-cp1-F and HDR1-cp1-R, S1 Table) and then cloned into the vector pCam2300K. The two constructs were introduced into *Agrobacterium tumefaciens* strain AGL1 by electroporation and then transformed into *hdr1* mutant calli. The regenerated plants were grown in a paddy field at the CAAS in Beijing, China [42]. The genotype of each transgenic plant was determined by PCR.

### Plasmid construction and generation of *p35S*::*HDR1*-*RNAi*, *p35S*::*OsK4-RNAi*, *p35S*::*OsK3*-*RNAi*, and *pActin1*::*Flag*:*HDR1* transgenic plants

DNA encompassing 113 bp of the 5' UTR of *HDR1*, 150 bp of the 5' UTR of *OsK4* and 160 bp of the 5' UTR of *OsK3* were amplified using specific primer pairs (S1 Table) and inserted into pTCK309 using the *Sac*I, *Spe*I, *Kpn*I, and *BamH*I sites in inverted orientations. The construct was transferred to *A*. *tumefaciens* strain AGL1 by electroporation and then introduced into WT rice calli. The regenerated plants were grown in a paddy field at the CAAS in Beijing. Early flowering RNAi transgenic plants were selected and analyzed. To generate *pActin1*::*Flag*:*HDR1*, the coding region of *HDR1* was amplified using a specific primer pair (S1 Table) and inserted into pCamabia1307 using the *Xba*I and *Sal*I sites. The plasmid containing *Flag-HDR1* was digested with *Xba*I and *Sal*I and then inserted into pCambia2300Actin1 using the *Xba*I and *Sal*I sites. The construct was introduced into *hdr1*calli through *A*. *tumefaciens*-mediated transformation, and the transgenic plants were grown in a paddy field and analyzed by comparing the flowering time with WT plants.

### Subcellular localization of HDR1 and OsK4

The full-length coding sequence of *Hdr1* or *OsK4* was PCR-amplified from cDNA using the primers pairs HDR1-GFP-fw and HDR1-GFP-rv or OsK4-GFP-fw and OsK4-GFP-rv (S1 Table) and cloned into pAN580 to generate *p35S*:: *HDR1*:*GFP* or *p35S*::*OsK4*:*GFP*. The fusion plasmid and pMcherry (*p35S*::*RFP*, as a control) were transiently co-expressed in rice leaf protoplasts by PEG (polyethylene glycol) as previously described [38]. The protoplast cell layers were then examined by laser scanning confocal microscopy [Leica TCS SP2; Wetzlar, Germany]. GFP fluorescence was imaged using excitation with the 488-nm argon laser line and a 505–530-nm band-pass emission filter.

### Reverse transcription (RT)-PCR and quantitative (q)RT-PCR

Total RNA was extracted using the RNeasy Plant Mini Kit (QIAGEN, Hiden, Germany) from various rice plants (WT, *hdr1*, *hd1*, *ehd1*, *OsK4-RNAi*, *OsK3*-*RNAi*, *HDR1*-overexpressing and various flowering relating genes [38]). For mutant or near isogenic lines of flowering time genes, penultimate leaves were harvested around the reported peak expression level of each gene during the 24-h photoperiod according to Gao (Penultimate leaves were harvested around the reported peak expression level of each gene during the 24-h photoperiod at dawn for *OsPhyB*, *Ehd1*, *Ehd2*, *Hd3a*, *RFT1* and *Ehd4*; 3 h after dawn for *Ghd7*; 8 h after dawn for *Ehd3*, *OsMADS50*, *OsMADS51* and *DTH8*; and immediately after dusk for *OsGI* and *Hd1* [38]). Total RNA (2 μg) from each sample was reverse-transcribed with an *oligo(dT)* primer and Ace RT Enzyme [Toyobo, Osaka, Japan] according to the manufacturer’s instructions. The PCR conditions were as follows: preincubation at 94°C for 2.5 min, followed by 30 cycles of 94°C for 20 s, 52°C for 20 s, and 72°C for 30 s. qRT-PCR was performed on an iQ5 Multicolor Real-Time PCR Detection System [Bio-Rad] with SYBR Green Real-Time PCR Master Mix (Life Technologies). The rice *Ubiquitin1* gene was selected as an internal standard. The fold change of the transcript level of a gene of interest to that of *UBQ1* is calculated as 2^-ΔCt^ [ΔCT = CT (gene of interest)—CT (UBQ)]. The primer pairs for other gene amplifications are specified in S2 Table.

### Histochemical staining for GUS activity

GUS assays were carried out as described previously [43]. Rice tissues were incubated in GUS staining solution (50 mM sodium phosphate, pH 7.0, 10 mM EDTA, 0.1% Triton X-100, 1 mg/ml X-gluc, 0.1 mM potassium ferricyanide, and 10% methanol) at 37°C for 10–12 h, followed by washing with 70% ethanol. The stained tissues were imaged under a zoom stereo microscope [Nikon SMZ1000; Tokyo, Japan].

### BiFC assay

The protein-coding regions of *HDR1* and *OsK4* were amplified using gene-specific primers (S1 Table). Then, the PCR products were ligated into pSAT1-nVenus-C and/or pSAT1-cCFP-N using the *EcoR*I and *Xho*I/*Sal*I sites to produce pSAT1-nVenus-C-HDR1, pSAT1-cCFP-N-OsK4, pSAT1-nVenus-C-OsK4, and pSAT1-cCFP-N-HDR1. All constructs were verified by sequencing. Plasmid pairs were transfected into *Arabidopsis* protoplasts as described previously, using pairs of each construct and the empty vector as negatives control [53]. The cells were then examined by laser scanning confocal microscopy (Leica TCS SP2). YFP fluorescence was imaged by excitation with the 515-nm argon laser line and a 535–565-nm band-pass emission filter.

### Yeast two-hybrid analysis

The protein-coding regions of *HDR1*, *OsK3*, *OsK4*, *Hd1*, *Ehd1*, *Hd3a*, *RFT1*, *Ehd2*, *Ehd3*, *Ehd4*, *OsMADS50*, *OsMADS51*, *OsGI*, *OsPhyB*, *DTH8 and Ghd7* were amplified using gene-specific primers (S1 Table). Then, the PCR products were fused into the activation domain (AD) vector pGADT7 and/or the DNA-binding domain (BD) vector pGBKT7 using the *EcoR*I and *Xho*I/*Sal*I sites. All constructs were verified by sequencing. The constructs were then transformed into *Saccharomyces cerevisiae* strain AH109 [BD Biosciences, Palo Alto, CA, USA] according to the manufacturer’s protocol. No detectable self-activation was observed for each construct on SD selective medium (SD-His-Leu + 5 mM 3-AT or SD-His-Trp + 5 mM 3-AT). Yeast cells were spotted on SD media lacking leucine, tryptophan, histidine and adenine.

### Yeast one-hybrid assay

The 2.2-kb and 4.2-kb promoter regions of *Hd1* and *Ehd1* were amplified (S1 Table), and the fragments were then cloned into the pLacZi vector using *EcoR*I and *Xho*I sites to generate *pLacZip*-*Hd1* and *pLacZip*-*Ehd1*. The resulting plasmids were used to transform yeast cells, followed by secondary transformation with *pGBKT7-HDR1* and selection according to the manufacturer’s manual [PT1031-1, CLONTECH Laboratories Inc., Mountain View, CA]. Full-length of *OsLFL1* cDNA was amplified (S1 Table) and then inserted into plasmid pGBKT to generate *pGBKT*-*OsLFL1* as a positive control, as OsLFL1 binds the *Ehd1* promoter region. Plasmids were co-transformed into the yeast strain YM4271. Yeast cell were spotted onto SD/His- plates and grown for 2 days. Then, the yeast cells were transferred onto SD/His-/Leu- 45mM 3-AT plates with X-gal (5-bromo-4-chloro-3-indolyl-b-D-galacto-pyranoside) for blue color detection.

### Yeast three-hybrid assay

*HDR1* was amplified (S1 Table) and digested with *EcoR*I and *BamH*I, *OsK4* was amplified and digested with *BamH*I and *Pst*I, and the PCR fragments were then introduced into the pBridge vector using the *EcoR*I and *Pst*I sites, generating *pBridgeHDR1-OsK4*. Then, the vector sequence was verified by DNA sequencing, and the constructs were used to transform yeast cells. The resulting strains were co-transformed with the AD fusion vector *pGADT7-HD1* and *pGADT7-EHD1*. Transformants were selected on medium according to the manufacturer’s instructions (PT3212-5; CLONTECH Laboratories Inc., Mountain View, CA, USA). Plasmids were co-transformed into the yeast strain AH109. Yeast cells were spotted and grown on SD medium without leucine, tryptophan, histidine and adenine.

### Western blotting

Total proteins were extracted from six-week-old rice seedlings in RIPA buffer (50 mM Tris-HCl, pH 7.2, 150 mM NaCl, 10 mM MgCl_2_, and 1 mM phenylmethylsulfonyl fluoride, protease inhibitor mixture [Sigma, St. Louis, MO, USA]), and the supernatant was collected. The total protein concentration was determined using a Bradford protein assay kit (Bio-Rad). Proteins were detected using anti-FLAG [Sigma, St. Louis, MO, USA], anti-HDR1, anti-HD1 or anti-OsK4 [kindly supplied by Dr. Liu, BIG, CAS] antibodies at 1:4000 dilution and visualized with an enhanced chemiluminescence (ECL) reagent (GE Healthcare).

The HIS-HDR1 fusion protein were overexpressed in *Escherichia coli* (strain BL21) and purified to generate multi-clone antibodies. Frozen cells were extracted with 100 μl of extraction buffer (50 mM Tris-HCl. pH 7.6, 15 mM MgCl_2_, 0.1 M KCl, 0.25 M sucrose, 10% glycerol, 1 mM phenylmethylsulfonyl fluoride, protease inhibitor mixture [Sigma, St. Louis, MO, USA], and 14 mM β-mercaptoethanol). After centrifugation of the sample at 15,000 x *g* for 10 min, the supernatant was sampled, and the protein concentration was measured. The amount of protein form each extract was measured according to the manufacturer’s instructions [Laboratories Inc., Mountain View, CA, USA].

### Co-immunoprecipitation

For Co-IP assays, total protein extracted from FLAG:HDR1 transgenic or none-transgenic and *OsK4-RNAi* transgenic or none-transgenic was mixed with purified 2mg HIS-HD1 and anti-FLAG antibody. The mixture was incubated overnight with Magna Protein A Magnetic Beads [Millipore, Temecula, California, USA] at 4°C in 1000 μl of binding buffer (20 mM Tris-HCl, pH 7.6, 2.5 mM β-mercaptoethanol, and 0.1 M NaCl). After incubation, the beads were washed five times with washing buffer (20 mM Tris-HCl, pH 7.5, 500 mM NaCl, and 0.5% Triton X-100). After washing, 40 μl of 1× SDS-PAGE sample buffer was added, the mixture was denatured, and the sample was loaded on a 10% SDS-PAGE gel. Proteins were detected using a horseradish peroxidase (HRP)-conjugated anti-FLAG [Sigma, St. Louis, MO, USA], anti-HD1 or anti-HIS antibody, and then visualized with an ECL reagent (GE Healthcare).

### Protein phosphorylation assay

Protein phosphorylation experiments were carried out based on previously described [54]. Briefly, for *in vitro* phosphorylation of rice HD1 by OsK4, purified GST-OsK4, FLAG-HD1 and/or HIS-HDR1 fusion proteins in reaction buffer (50 mM HEPES-KOH (pH 7.5), 10 mM MgCl_2_, 5 mM MnCl_2_, 1 mM DTT, 0.1 mM ATP, and 10 mCi of [γ-^32^P]ATP). The mixture was allowed to react for 30 min at 30°C. The phosphorylation of proteins was taken autoradiogram using a phosphor imager after SDS-PAG electrophoresis.

Immunoprecipitated proteins from *FLAG-HDR1* plant bound to the protein A beads were also subjected to kinase assays. Each reaction contained 10μl of beads in reaction buffer as described above. The mixture was allowed to react for 30 min at 30°C. Radioactive signals were detected using a phosphor imager. An HD1 phosphorylation assay was also conducted using anti-HIS antibodies.

## Supporting Information

S1 Fig *HDR1* overexpression does not promote Nipponbare flowering. (A) Phenotype of *HDR1* overexpression plants under LDs. (B) The dates of flowering are unchanged for WT and Overexpression plants (OX) under NDs, LDs and SDs. (C) The expression patterns of the key flowering-related genes (*Hd1*, *Ehd1*, *Hd3a* and *RFT1*) are similar in WT and OX plants, as determined by semi-quantitative RT-PCR.
(TIF)

S2 Fig The C-terminus of HDR1-like family proteins are highly conserved. (TIF)

S3 Fig The rhythmic expression pattern of *HDR1* in the *hd1* and *ehd1* mutants under SD and LD (A), and the expression levels of *OsGI*, *OsPhyB*, *Ehd2*, *Ehd3*, *Ehd4*, *Ghd7*, *DTH8*, *OsMADS50*, and *OsMADS51* in the WT and *hdr1* under LDs (B).
(TIF)

S4 Fig HDR1 does not interact with known flowering-regulation proteins in the Y2H system. (A) HDR1 or OsK4 had no interaction with HD1 and EHD1. (B) HDR1 had no association with HD3A, RFT1, EHD2, EHD3, EHD4, OsGI, OsPHYB, OsMADS50, OsMADS51 and DTH8.
(TIF)

S5 Fig OsK3 shares high sequence identity with OsK4 and interacts with HDR1. (A) Sequence alignment of OsK3 and OsK4. (B) OsK3 physically interacted with HDR1 in yeast.
(TIF)

S6 Fig *hdr1* exhibits late germination, as reported for *osk3* and *osk4*. (TIF)

S7 Fig Specificity of the antibody for the detection of OsK4 (approximately 56 kD) as determined by Western blotting. Proteins were exacted from 6-week-old WT, *OsK3-RNAi* and *OsK4-RNAi* leaves.
(TIF)

S8 Fig HDR1 does not bind the promoter regions of *Hd1* and *Ehd1*. The flowering **regulator** OsLFL1 was used as a positive control.
(TIF)

S9 Fig HDR1 or OsK4 localizes to the nucleus in the *OsK4*-RNAi and *hdr1*. (A)-(D) The location of OsK4 was not changed in *hrd1*. A, HDR1-GFP; B, Bright field; C, *OsMADS3*-mCherry; and D, Merged. (E) to (H) The location of HDR1 was not changed in the *OsK4-RNAi-7* transgenic line. E, HDR1-GFP; F, Bright field; G, *OsMADS3*-*mCherry*; and H, Merged. (I) to (L) The location of HDR1 was not changed in the *OsK4-RNAi-19* transgenic line. I, HDR1-GFP; J, Bright field; K, *OsMADS3*-*mCherry*; and L, Merged. Bar = 10μm.
(TIF)

S10 Fig *pActin1*::*FLAG-HDR1* fully rescues the early flowering of *hdr1* phenotype. (TIF)

S1 Table Primers used for plasmids construction. (DOC)

S2 Table Primers used for real-time quantitative PCR and semi-quantitative RT-PCR. (DOC)

S3 Table The flowering times of transgenic line for *hdr1* complementation. (DOC)
