# Supplementary figures and images for: The Oryza sativa Regulator HDR1 Associates with the Kinase OsK4 to Control Photoperiodic Flowering
Source: PLoS Genet. 2016 Mar 8;12(3):e1005927. doi: 10.1371/journal.pgen.1005927 (PMC4783006; doi:10.1371/journal.pgen.1005927)

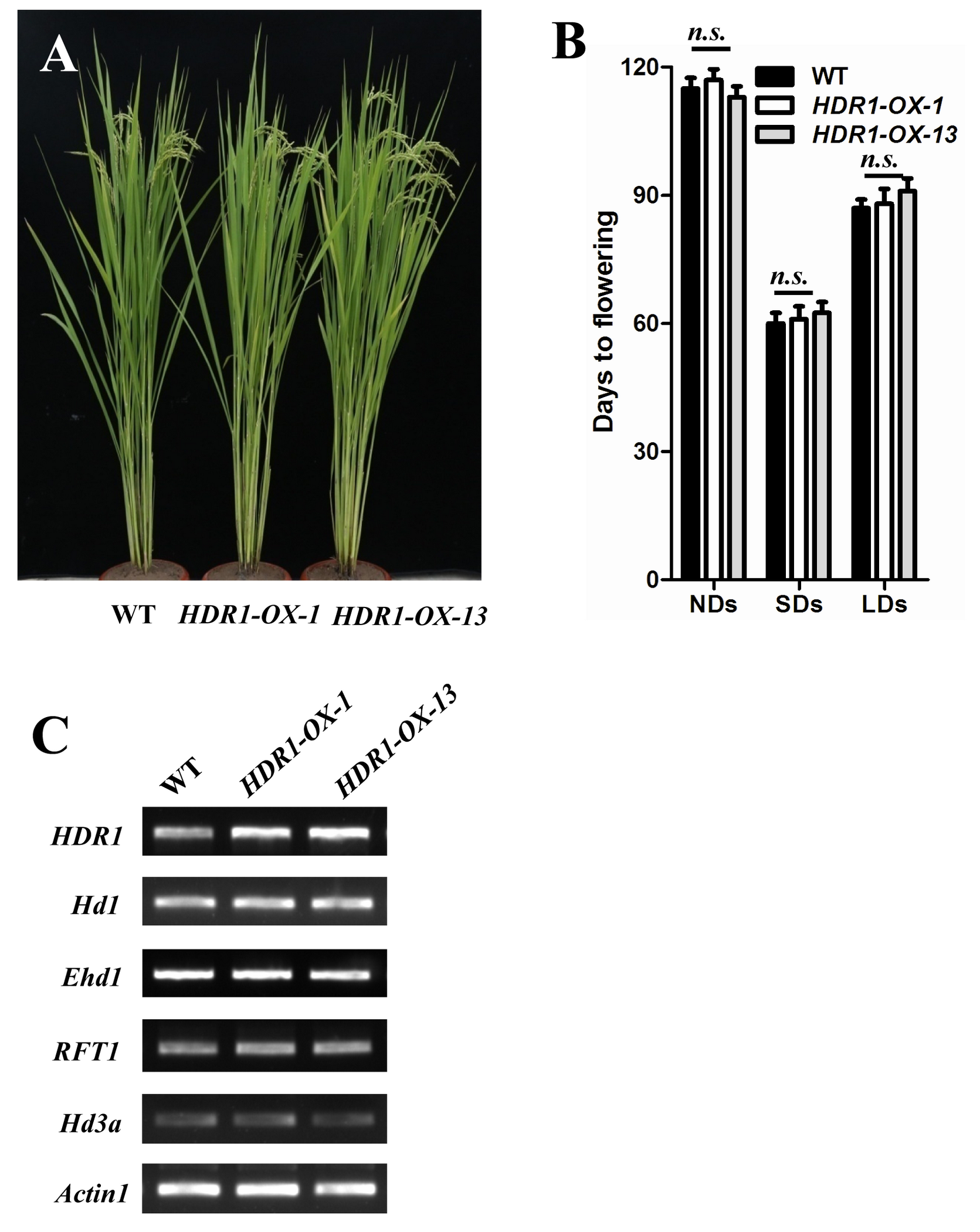

Supplement: S1 Fig — (A) Phenotype of HDR1 overexpression plants under LDs. (B) The dates of flowering are unchanged for WT and Overexpression plants (OX) under NDs, LDs and SDs. (C) The expression patterns of the key flowering-related genes (Hd1, Ehd1, Hd3a and RFT1) are similar in WT and OX plants, as determined by semi-quantitative RT-PCR. (TIF) [file pgen.1005927.s001.tif]

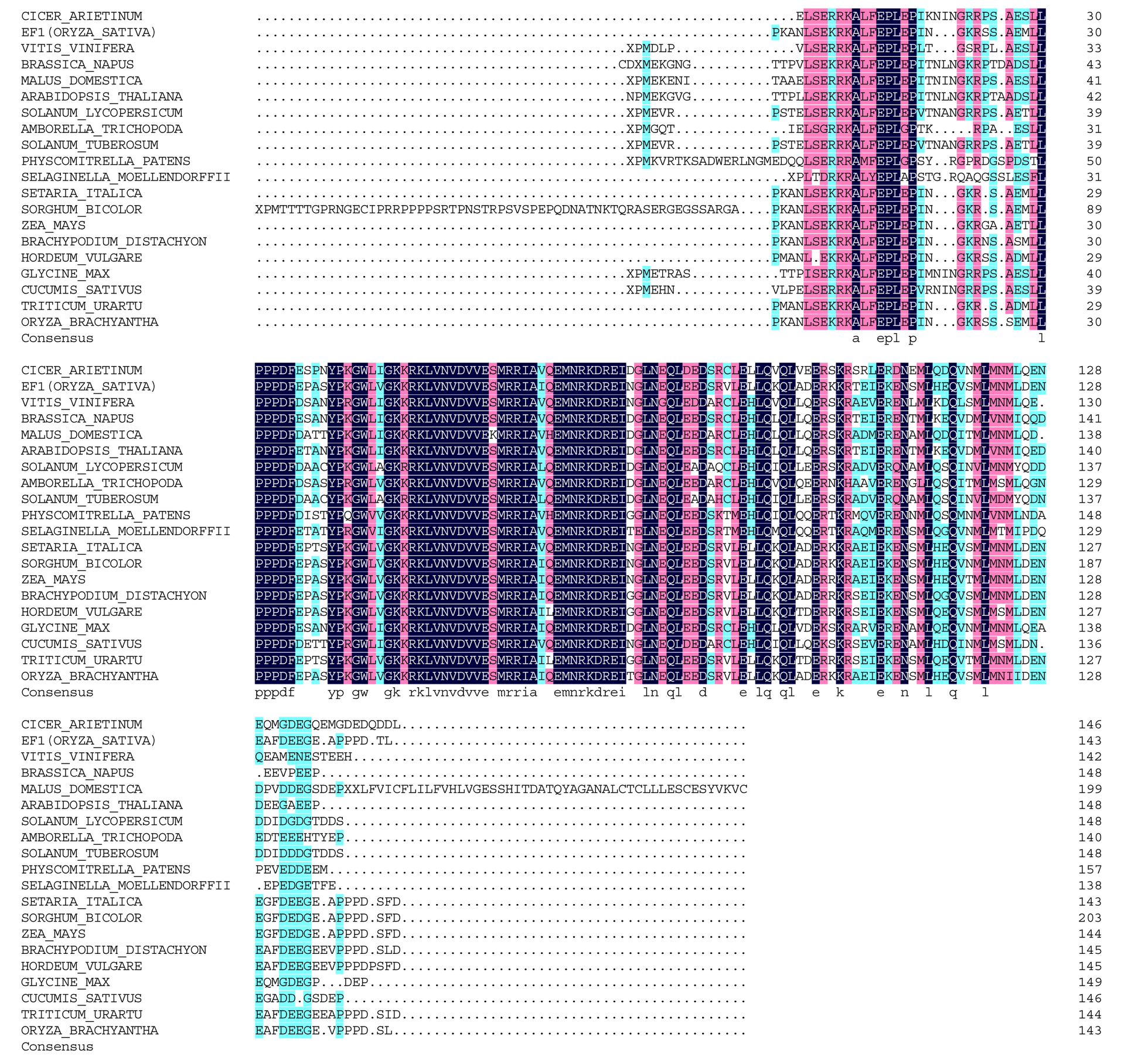

Supplement: S2 Fig — (TIF) [file pgen.1005927.s002.tif]

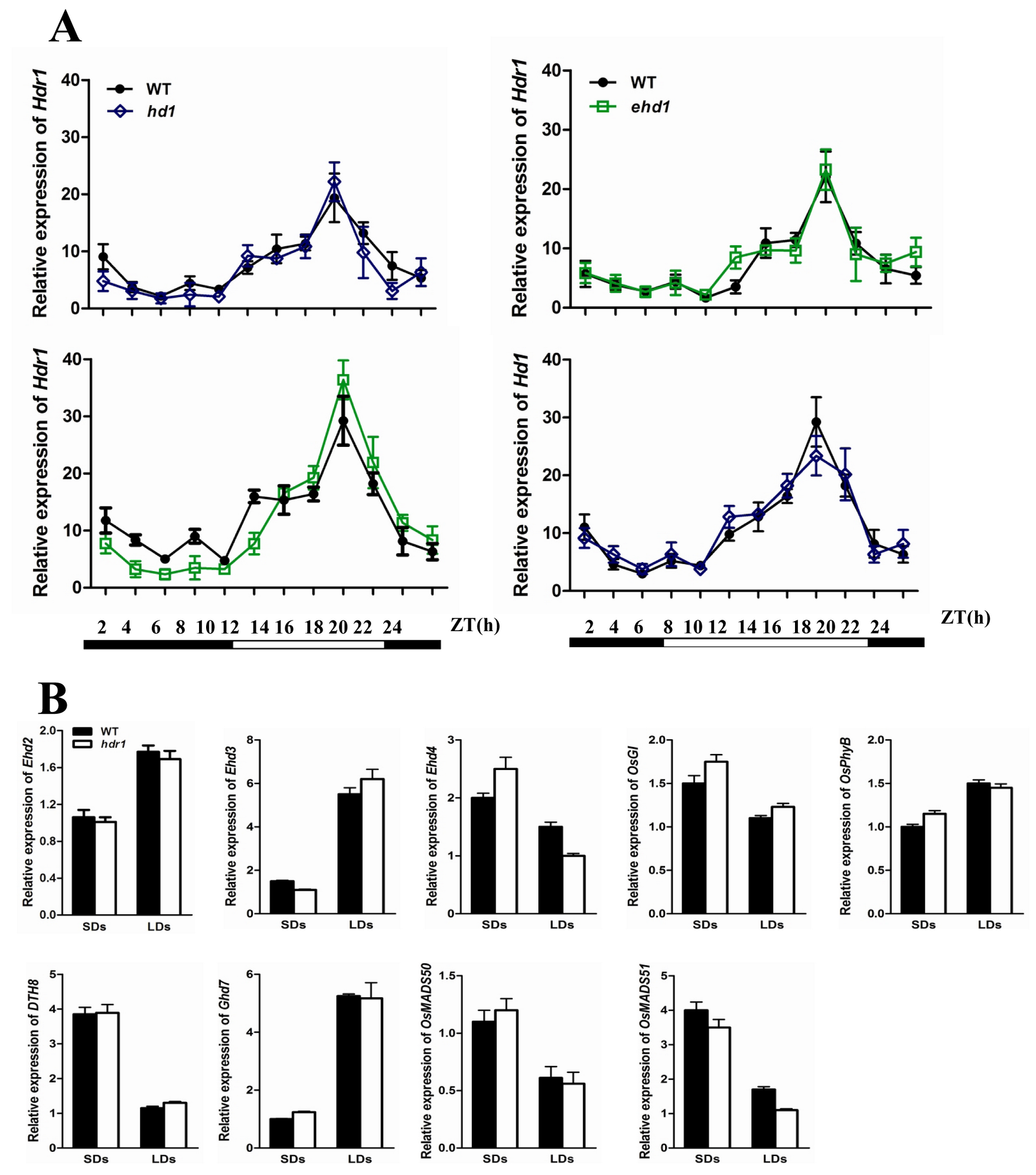

Supplement: S3 Fig — The rhythmic expression pattern of HDR1 in the hd1 and ehd1 mutants under SD and LD (A), and the expression levels of OsGI, OsPhyB, Ehd2, Ehd3, Ehd4, Ghd7, DTH8, OsMADS50, and OsMADS51 in the WT and hdr1 under LDs (B). (TIF) [file pgen.1005927.s003.tif]

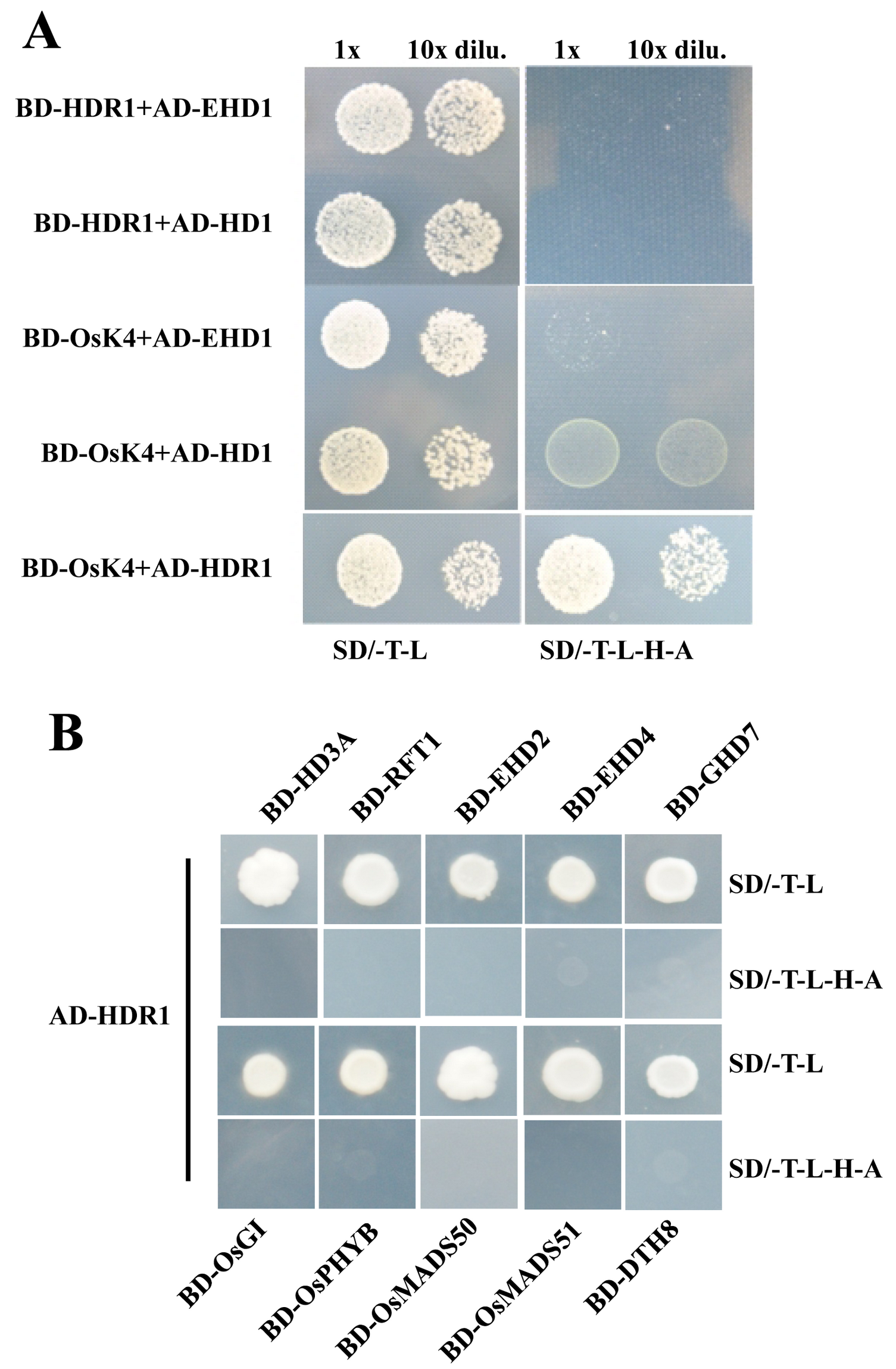

Supplement: S4 Fig — (A) HDR1 or OsK4 had no interaction with HD1 and EHD1. (B) HDR1 had no association with HD3A, RFT1, EHD2, EHD3, EHD4, OsGI, OsPHYB, OsMADS50, OsMADS51 and DTH8. (TIF) [file pgen.1005927.s004.tif]

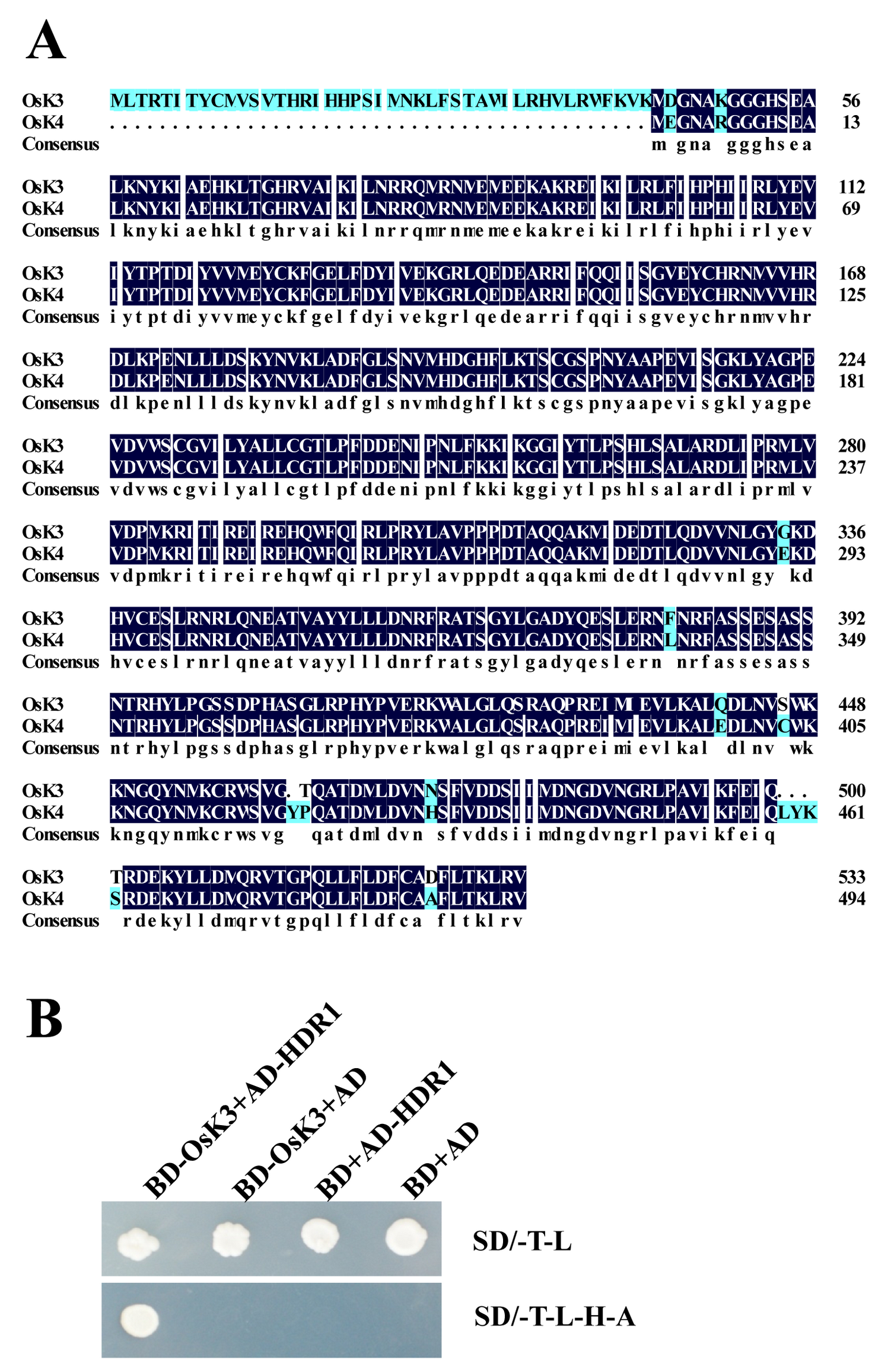

Supplement: S5 Fig — (A) Sequence alignment of OsK3 and OsK4. (B) OsK3 physically interacted with HDR1 in yeast. (TIF) [file pgen.1005927.s005.tif]

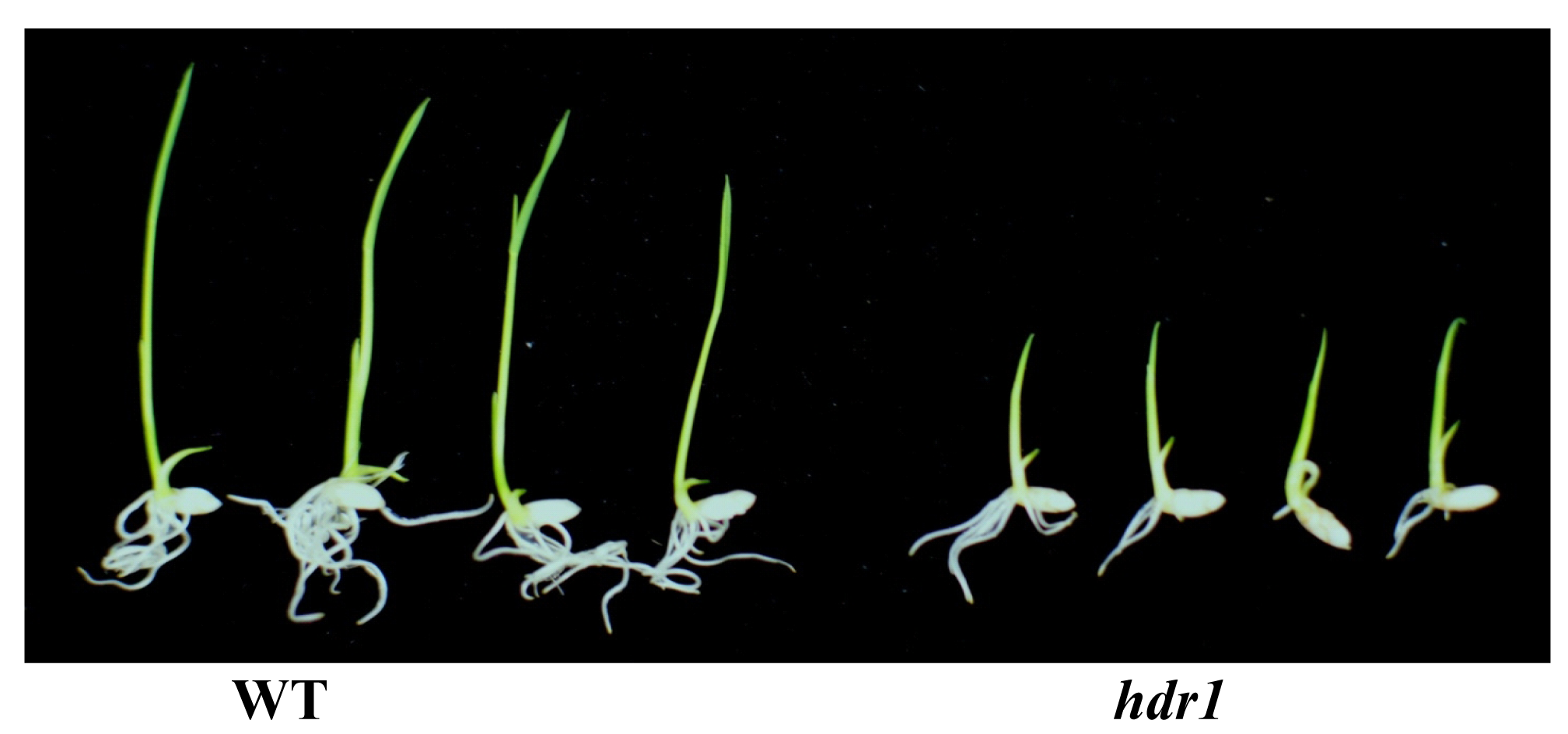

Supplement: S6 Fig — (TIF) [file pgen.1005927.s006.tif]

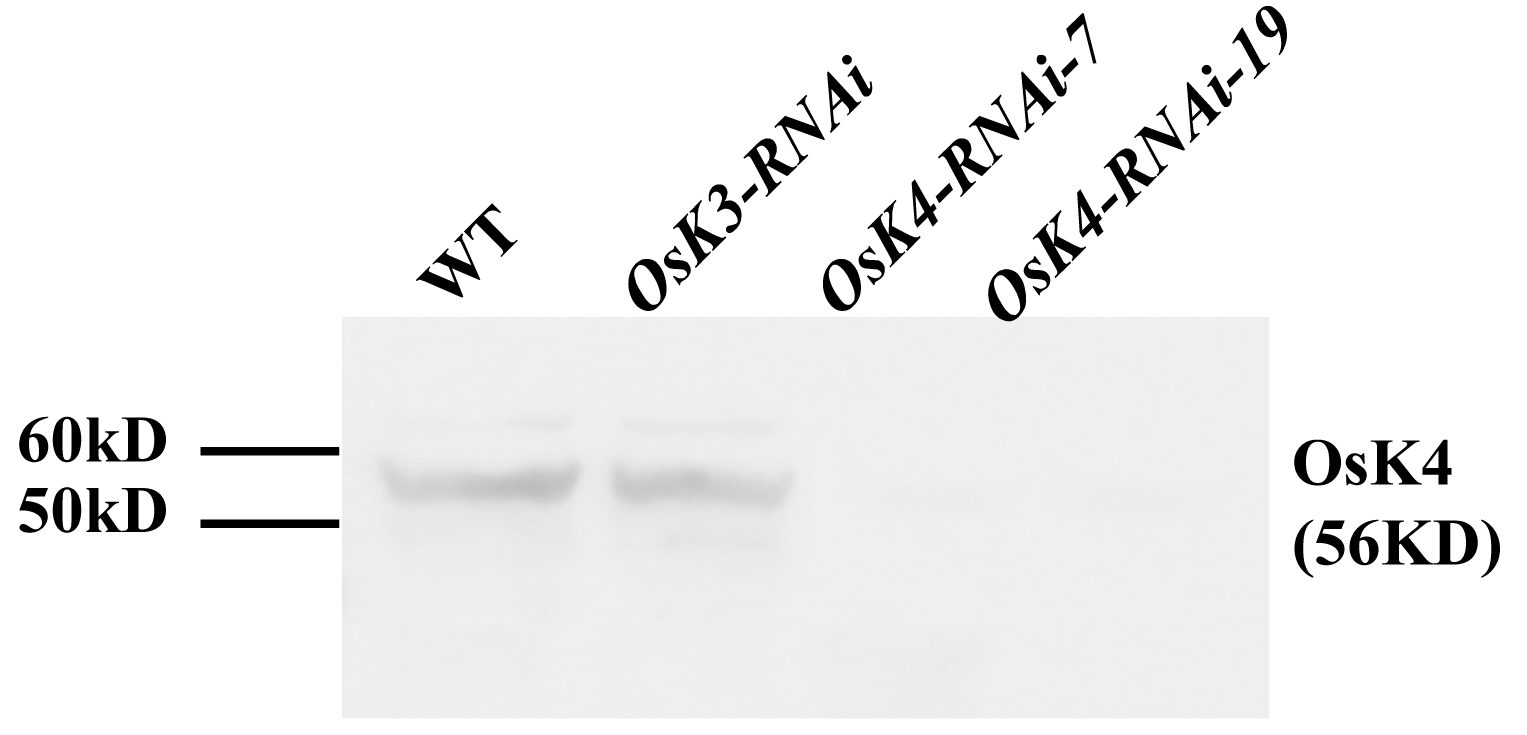

Supplement: S7 Fig — Proteins were exacted from 6-week-old WT, OsK3-RNAi and OsK4-RNAi leaves. (TIF) [file pgen.1005927.s007.tif]

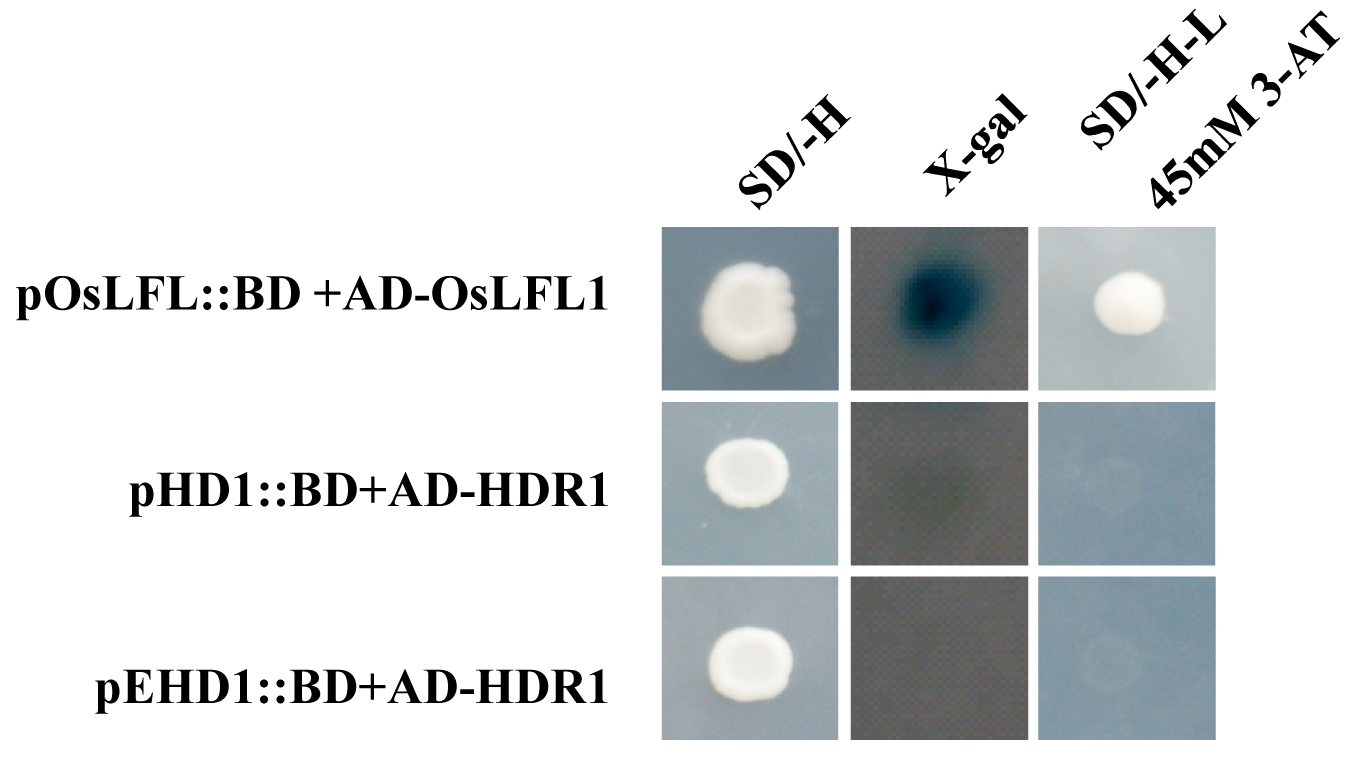

Supplement: S8 Fig — The flowering regulator OsLFL1 was used as a positive control. (TIF) [file pgen.1005927.s008.tif]

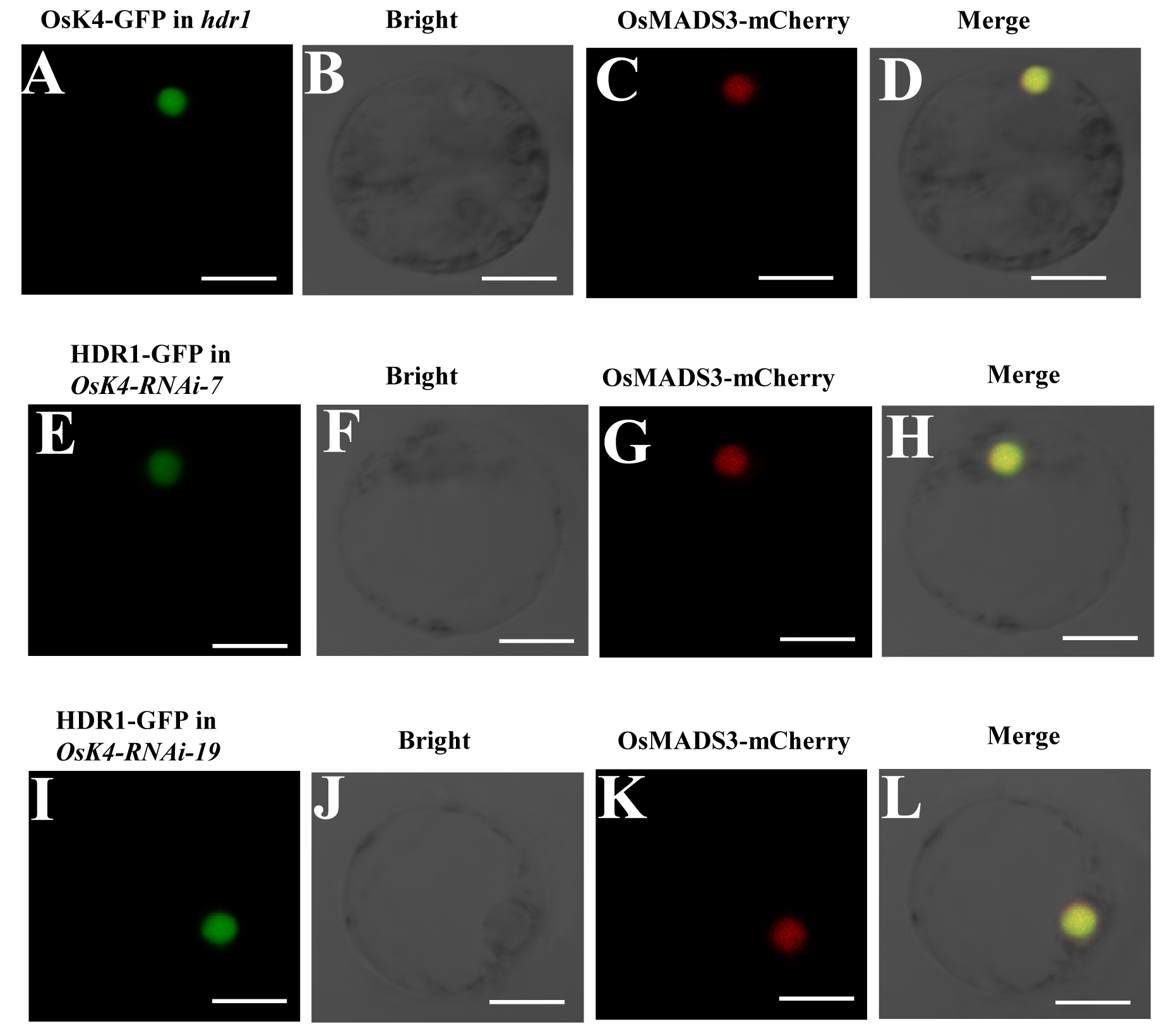

Supplement: S9 Fig — (A)-(D) The location of OsK4 was not changed in hrd1. A, HDR1-GFP; B, Bright field; C, OsMADS3-mCherry; and D, Merged. (E) to (H) The location of HDR1 was not changed in the OsK4-RNAi-7 transgenic line. E, HDR1-GFP; F, Bright field; G, OsMADS3-mCherry; and H, Merged. (I) to (L) The location of HDR1 was not changed in the OsK4-RNAi-19 transgenic line. I, HDR1-GFP; J, Bright field; K, OsMADS3-mCherry; and L, Merged. Bar = 10μm. (TIF) [file pgen.1005927.s009.tif]

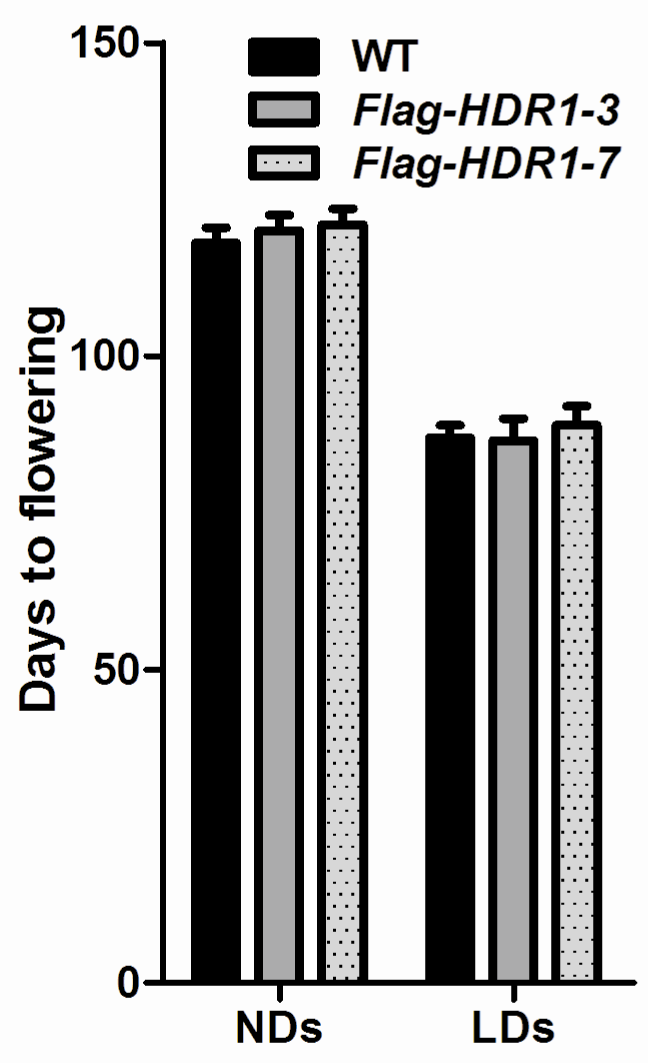

Supplement: S10 Fig — (TIF) [file pgen.1005927.s010.tif]
