# Supplementary material for: The Oryza sativa Regulator HDR1 Associates with the Kinase OsK4 to Control Photoperiodic Flowering
Source: PLoS Genet. 2016 Mar 8;12(3):e1005927. doi: 10.1371/journal.pgen.1005927 (PMC4783006; doi:10.1371/journal.pgen.1005927)
Supplement: S1 Table — (DOC) [file pgen.1005927.s011.doc]

S1 Table. Primers used for plasmids construction.

| **Primer name** | **Sequences of primer** | **Remarks** |
| --- | --- | --- |
| Hdr1-CP1-F | ACTAGT CTGGGTCAATTATGGCTCAAGTC | KpnI |
| Hdr1-CP1-R | CTCGAG CAGAGGATTTCGATGACTGAAG | XhoI |
| Hdr1-CP2-R | CTCCTCCATGGCTTGCTCTTGC | NcoI |
| Hdr1-CP3-F | GCAAGAGCAAGCCATGGAGGAG | NcoI |
| Hdr1-CP3-R | CTCGAG TGGTATCCGAAGATGTGTAG | XhoI |
| Hdr1-GFP-F | TCTAGA GAGGAGGAGCAAGAGCAAG | XbaI |
| Hdr1-GFP-R | CCCGGG TAAGGTATCAGGTGGTGGTG | SmaI |
| Hdr1-RNAi-F | GAGCTCGGATCC ATAGAGAGAGGGACAGAGAGAGAG | SacI-BamHI |
| Hdr1-RNAi-R | ACTAGTGGTACC CTCCTCCTCCTCCTTCACTCTTC | KpnI-SpeI |
| OsK3-RNAi-F | GAGCTCGGATCC GATGGTGCCAATGCAGGATG | SacI-BamHI |
| OsK3-RNAi-R | ACTAGTGGTACC GTGCAGTAGAAGCAGAGTTAACG | KpnI-SpeI |
| OsK4-RNAi-F | GAGCTCGGATCC GTCGTCCTGCTGCTTCC | SacI-BamHI |
| OsK4-RNAi-R | ACTAGTGGTACC AGTGACCAGGATGGATGC | KpnI-SpeI |
| Hdr1-BD-F | GAATTC GAGGAGGAGCAAGAGCAAG | EcoRI |
| Hdr1-BD-R | GGATCC gTGGTATCCGAAGATGTGTAG | BamHI |
| Hdr1-AD-F | GAATTC GAGGAGGAGCAAGAGCAAG | EcoRI |
| Hdr1-AD-R | GGATCC gTGGTATCCGAAGATGTGTAG | BamHI |
| Hdr1-Bridge-F | GAATTC GAGGAGGAGCAAGAGCAAG | EcoRI |
| Hdr1-Bridge-R | GGATCC gTGGTATCCGAAGATGTGTAG | BamHI |
| OsK3-BD-F | GAATTC GTGAACATGTTAACTCGAAC | EcoRI |
| OsK3-BD-R | GGATCC gTAGAACCCTCAGCTTGGTAAG | BamHI |
| OsK4-BD-F | GAATTC ATGGAAGGAAATGCTAGAGG | EcoRI |
| OsK4-BD-R | GGATCC gTAGAACCCTCAGTTTGGTAAGG | BamHI |
| OsK4-Bridge-F | GGATCC ggATGGAAGGAAATGCTAGAGG | BamHI |
| OsK4-Bridge-R | CTGCAG gTAGAACCCTCAGTTTGGTAAGG | PstI |
| Hd1-1h-F | GAATTC GTACCATACTCTGCACCTCAC | EcoRI |
| Hd1-1h-R | CTCGAG CCTGGTCGAACACGTTGC | XhoI |
| Ehd1-1h-F | GAATTC GAACGTAGTATCTGACTGATACG | EcoRI |
| Ehd1-1h-R | CTCGAG ACCAGAACTCTTAGTCCATAAGG | XhoI |
