# Supplementary material for: The Oryza sativa Regulator HDR1 Associates with the Kinase OsK4 to Control Photoperiodic Flowering
Source: PLoS Genet. 2016 Mar 8;12(3):e1005927. doi: 10.1371/journal.pgen.1005927 (PMC4783006; doi:10.1371/journal.pgen.1005927)
Supplement: S2 Table — (DOC) [file pgen.1005927.s012.doc]

S2 Table. Primers used for real-time quantitative PCR and semi-quantitative RT-PCR.

| **Primer name** | **Forward (5’-3’)** | **Reverse (5’-3’)** |
| --- | --- | --- |
| *Ubq* | GCTCCGTGGCGGTATCAT | CGGCAGTTGACAGCCCTAG |
| *Hd3a* | GCTCACTATCATCATCCAGCATG | CCTTGCTCAGCTATTTAATTGCATAA |
| *RFT* | TGACCTAGATTCAAAGTCTAATCCTT | TGCCGGCCATGTCAAATTAATAAC |
| *Hd1* | TCAGCAACAGCATATCTTTCTCATCA | TCTGGAATTTGGCATATCTATCACC |
| *Ehd1* | CCTACAGTGATTATGGCTTCA | GTGCTGCCAAATGTTGCTC |
| *Ehd2* | CGACGACAATAGCTCGATCGC | GTGCATGGTCACGGAGCCTT |
| *Ehd3* | GACCACCTCGTCACCTACAAG | GAGTGTCCCTCCAGCTAATCC |
| *Ehd4* | CAGCCAGCGGAATCATCAC | CCAAATCCATCAGACCTACTCCT |
| *MADS50* | ATGCAATGACACCAAACCATC | GGTAGTGGAGTCTGCCGATC |
| *MADS51* | TCCTGTGCATAAGTTTGGCAGT | TCTACCTGGGATCAATCAGTGG |
| *DTH8* | CAGGAGTGCGTGTCGGAGTT | GGTCGTCGCCGTTGATGGT |
| *Ghd7* | GCTTGAACCCAAACACGG | CTCATCTCGGCATAGGCTT |
| *OsPhyB* | CATGCGTTGATTGGTTGTGAAG | ATCCTCCTCCTTTGGCACTCTTC |
| *Ef1*(Q-p1/ Q-p2) | GATAGAGAGAGGGACAGAGAGAGAG | CTCCTCCTCCTCCTTCACTCTTC |
| *Ef1*(Q-p3/ Q-p4) | CAGTAAGTACTGACGACGATGTTC | CTTGCCGTTGATTGGTTCCAAAG |
