# Supplementary material for: The Oryza sativa Regulator HDR1 Associates with the Kinase OsK4 to Control Photoperiodic Flowering
Source: PLoS Genet. 2016 Mar 8;12(3):e1005927. doi: 10.1371/journal.pgen.1005927 (PMC4783006; doi:10.1371/journal.pgen.1005927)
Supplement: S3 Table — (DOC) [file pgen.1005927.s013.doc]

**Table S3. Numbers of transgenic line for *hdr1* complementation according to flowering time.**

| **Plants** | **Days to flowering** | **Numbers/ Numbers of lines** |
| --- | --- | --- |
| WT | 116±2 | 20 |
| *hdr1* | 88±2 | 20 |
| CP | 116±2 | 77 |
| CP | 112±2 | 44 |
| CP | 108±2 | 21 |
| CP | 104±2 | 12 |
| CP | 100±2 | 9 |
| CP | 96±2 | 18 |
| CP | 94±2 | 4 |
| CP | 90±2 | 3 |
| CP | 88±2 | 16 |
